# Defining human cardiac transcription factor hierarchies using integrated single-cell heterogeneity analysis

**DOI:** 10.1038/s41467-018-07333-4

**Published:** 2018-11-21

**Authors:** Jared M. Churko, Priyanka Garg, Barbara Treutlein, Meenakshi Venkatasubramanian, Haodi Wu, Jaecheol Lee, Quinton N. Wessells, Shih-Yu Chen, Wen-Yi Chen, Kashish Chetal, Gary Mantalas, Norma Neff, Eric Jabart, Arun Sharma, Garry P. Nolan, Nathan Salomonis, Joseph C. Wu

**Affiliations:** 1 Stanford Cardiovascular Institute, Stanford University, Stanford, CA 94305 USA; 2 Institute of Stem Cell Biology and Regenerative Medicine, Stanford University, Stanford, CA 94305 USA; 3 Departments of Medicine and Radiology, Stanford University, Stanford, CA 94305 USA; 4 Department of Cellular and Molecular Medicine, University of Arizona, Tucson, AZ 85724 USA; 5 Department of Bioengineering, Stanford University, Stanford, CA 94305 USA; 6 Division of Biomedical Informatics, Cincinnati Children’s Hospital Medical Center, Cincinnati, OH 45229 USA; 7 Baxter Laboratory for Stem Cell Biology, Department of Microbiology and Immunology, Stanford University School of Medicine, Stanford, CA 94305 USA; 8 Zephyrus Biosciences, Inc. 820 Heinz Ave, Berkeley, CA 94710 USA; 9 Department of Biochemistry, Stanford University, Stanford, CA 94305 USA

## Abstract

Human induced pluripotent stem cell-derived cardiomyocytes (hiPSC-CMs) have become a powerful tool for human disease modeling and therapeutic testing. However, their use remains limited by their immaturity and heterogeneity. To characterize the source of this heterogeneity, we applied complementary single-cell RNA-seq and bulk RNA-seq technologies over time during hiPSC cardiac differentiation and in the adult heart. Using integrated transcriptomic and splicing analysis, more than half a dozen distinct single-cell populations were observed, several of which were coincident at a single time-point, day 30 of differentiation. To dissect the role of distinct cardiac transcriptional regulators associated with each cell population, we systematically tested the effect of a gain or loss of three transcription factors (*NR2F2*, *TBX5*, and *HEY2*), using CRISPR genome editing and ChIP-seq, in conjunction with patch clamp, calcium imaging, and CyTOF analysis. These targets, data, and integrative genomics analysis methods provide a powerful platform for understanding in vitro cellular heterogeneity.

## Abstract (web summary)

Human induced pluripotent stem cell derived cardiomyocytes are a powerful model for cardiogenesis and disease in vitro. Here the authors comprehensively map cardiac differentiation using multiple modalities, including single-cell RNA seq and CyTOF, in cells with a gain  or loss of function in key cardiac transcription factors.

## Introduction

Heart tissue regeneration is a persistent challenge in the cardiovascular community. After a typical myocardial infarction, it is estimated that over 1 billion cardiomyocytes die^1^, leading to a drastic decline in heart function. Replacement of an infarcted myocardium with cardiomyocytes developed in vitro may be a viable solution. With continuing improvements in developing efficient differentiation, large quantities of highly purified populations of human embryonic stem cell-derived cardiomyocytes (hESC-CMs) or human induced pluripotent stem cell-derived cardiomyocytes (hiPSC-CMs) can now be obtained. However, these cardiomyocytes do not match their mature in vivo derived counterpart^2,3^, as they display differences in electrophysiological parameters^4–6^, contraction^5,7^, and gene expression profiles of nodal, atrial, and ventricular cardiomyocytes^8^. Hence, characterization and understanding of signaling pathways in cardiomyocyte development must be further improved to realize the full potential of hiPSC-CMs.

Modeling cardiovascular diseases, screening for drug toxicities, and understanding heart developmental processes all require a highly uniform population of cardiomyocytes. A complete transcriptome analysis could provide pertinent information on the maturation state of the cardiomyocytes, as well as which specific population of cardiomyocytes is being assessed. A critical bottleneck in understanding the whole transcriptome of hiPSCs is the lack of reference gene expression signatures for hiPSC-CMs. Differences in cardiomyocyte differentiation protocols, time points analyzed, media compositions, or purity of cardiomyocytes formed could all contribute to variable transcriptome interpretations. In addition, profiling cardiomyocytes by RNA-seq or microarray analysis on a bulk culture of cardiomyocytes (i.e., large cell numbers pooled together) only provides the average gene expression and a collapsed view of the splicing events present in cardiomyocytes. Recent analyses of single-cell cardiac heterogeneity in embryonic hearts provide exciting insights into a more complex mixture of cardiomyocyte populations and transitioning transcriptional programs emerging throughout development^9,10^. Similar analyses in human developing cardiomyocytes that could be linked to distinct functional properties of those cell states would shed significant light on the developmental and functional properties of distinct cardiomyocyte populations.

Here we identified subpopulations of hiPSC-CMs represented by distinct gene expression profiles through the extensive analysis of multiple orthogonal single-cell and bulk RNA-seq datasets. These cardiomyocyte populations were enriched for expression of the transcription factors *ISL1, NR2F2, TBX5, HEY2*, or *HOPX*. By combining RNA-seq, ChIP-seq, calcium imaging, electrophysiological analysis, CyTOF, and RNA-seq of cardiomyocytes deficient in three transcription factors, we were able to derive an integrated transcriptional regulatory model that implicates distinct hiPSC-CM states in cardiac maturation and physiology. These data demonstrate that in using the small molecule Wnt modulatory differentiation protocol, earlier hiPSC-CMs exhibit more atrial-like gene expression profiles while prolonged culture produces more ventricular-like gene expression and physiological signatures.

## Results

### Emerging cellular heterogeneity through differentiation

**Fig. 1:**
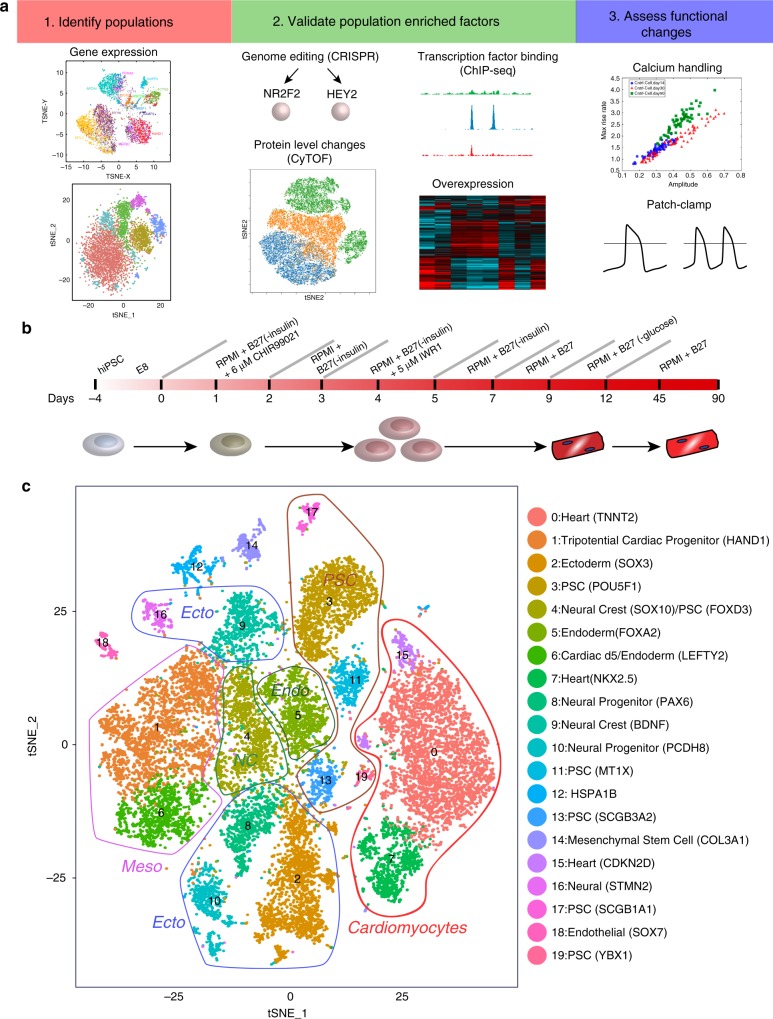
Identification of hiPSC-CM subpopulations by single-cell RNA-seq. **a** Integrative analytical workflow for identifying distinct cellular populations from single-cell RNA-seq analyses. Associated signatures can subsequently be further interrogated using orthogonal single-cell and bulk techniques (genome editing, ChIP-seq targets, overexpression models, changes at the protein level) and functional assays (calcium handling, patch-clamp). **b** A monolayer cardiomyocyte differentiation protocol was used to assess how single-cell populations change over time. Single-cell RNA-seq was performed on day 0, day 5, day 14, and day 45 of differentiation and 10,376 cell states identified using the software ICGS and Seurat-CCA. **c** A t-SNE plot was generated using AltAnalyze to visualize the expression of ICGS associated markers during the different days of differentiation. Twenty distinct cell populations were observed and could be attributed to populations representing pluripotent stem cells (PSC), definitive endoderm (DE), mesoderm (MESO), ectoderm (ECTO), stromal, neural crest (NC), endothelial cells, and early, mid or late cardiomyocyte progenitors

To define multiple potential sources of cellular heterogeneity in cardiomyocytes, we developed an integrated profiling strategy to compare in vitro derived cardiomyocytes to in vivo adult analogues, assessed their cellular and molecular heterogeneity, and associated these cell states with specific transcriptional regulators and physiological consequences (Fig. 1a). For these in vitro studies, we utilized a popular cardiomyocyte differentiation protocol^11^ where a heterogeneous population of cardiomyocytes (nodal-like, atrial-like, and ventricular-like) have been reported (Fig. 1b)^8^. To broadly define the cellular heterogeneity arising from this small molecule-based approach, we performed single-cell RNA-seq on a total of 10,376 cells from two hiPSC lines at 0, 5, 14, and 45 days of cardiac differentiation using droplet-based single cell sequencing (10X Chromium platform). Unsupervised delineation of cell states using the recently described Iterative Clustering and Guide-gene Selection (ICGS) algorithm identified 20 predominant cell states, containing distinct cardiac progenitor and heart-associated cell populations (Fig. 1c)^12^. Seurat-CCA and AltAnalyze also identified a number of distinct stem-progenitor populations- pluripotent stem cells (PSC), definitive endoderm (DE), mesoderm (MESO), ectoderm (ECTO), stromal, neural crest (NC), endothelial, and early, mid or late cardiomyocyte progenitors.

**Fig. 2:**
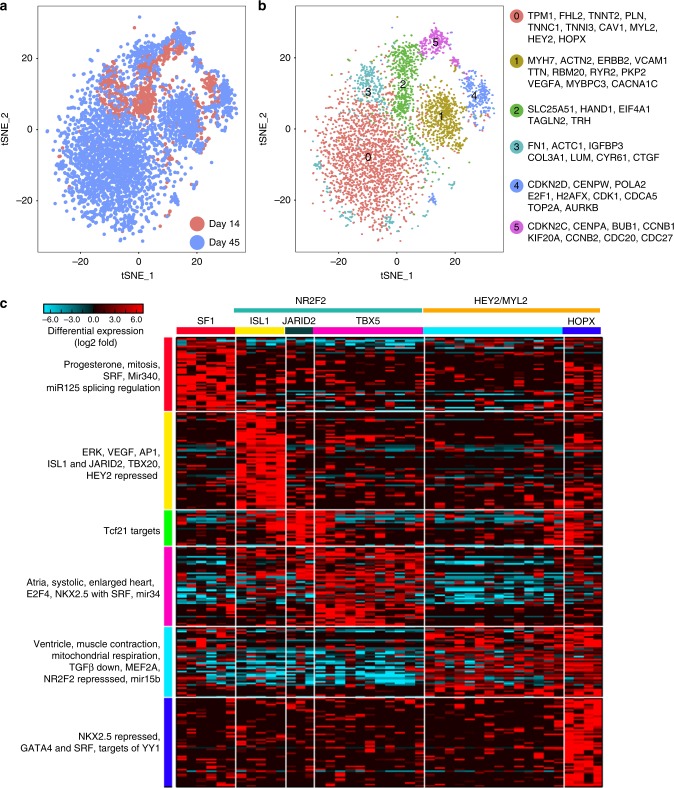
Single-cell RNA-seq identified subpopulations of cardiomyocytes. **a** Late stage cardiomyocytes (4,689 cells with *TNNT2* and *ACTC1* expression from day 14 and day 45) were further resolved using ICGS to identify subpopulations of cardiomyocytes. Associated t-SNE cell populations were colored by day of differentiation, as well as by cluster identified (markers within each cluster beside each cluster). **b** Six cardiomyocyte populations were identified representing subpopulations of cardiomyocytes in early proliferative stages (Cluster 4, 5) expressing cyclins, mid-cardiomyocyte stage (Cluster 2, 3) expressing *HAND1*, and more mature cardiomyocytes (Cluster 0, 1) expressing sarcomeric (*MYH6, MYL2, TNNT2, MYBPC3*) and calcium handling genes (*RYR2, PLN*). **c** Single cells from day 30 of differentiation were profiled using an independent technology (Fluidigm C1) to resolve coincident mid-to-late state differentiation heterogeneity. Genes associated with distinct observed cell populations identified by the algorithm MarkerFinder are shown. Factors with defining expression in each of the HOPACH clustered populations are shown (*SF1, ISL1, JARID2, TBX5, MYL2/HEY2*, and *HOPX)*. Two major groups in hiPSC-CMs were observed to express either *NR2F2* or *MYL2/HEY2*. Within each group, identified heart developmental regulators were identified using the software GO-Elite (left panel)

While this analysis identified multiple intriguing novel cell states at each time point of differentiation that appear transcriptionally distinct from cardiomyocytes, we chose to focus further on the heterogeneity within the cardiomyocyte population by isolating cardiac marker (*TNNT2* and *ACTC1*) expressing cells (4,689 cells) from day 14 and day 45 time points (Fig. 2a). A focused analysis of these maturing cardiomyocyte populations identified subpopulations of cardiomyocytes in early proliferative stages (Cluster 4, 5), mid-cardiomyocyte stage (Cluster 2, 3), and more mature stages (Cluster 0, 1) (Fig. 2b). Specifically, comparing cardiomyocytes from day 14 to those from day 45 revealed that early stage cardiomyocytes were enriched for the expression of *VCAM1*, *APOE, NPPA, NPPB, MYH6, COL2A1, HMGA1, S100A10, ATF4*, *RBFOX2*, *HMGA2*, and *CLDN6*, while the expression of *HEY2, FHL2, CAV1, SERPINI1, MYL2, A2M, LGALS3BP, TMEM173*, *SYNE2*, *NFIA*, and *HOPX*  were enriched in day 45 cardiomyocytes. Hence, we find a continuum of transitional transcriptomic hiPSC-CM gene expression states associated with maturity over time in cell culture (Supplementary Fig.1E).

### Transcriptional regulators and cardiac heterogeneity

These broad single-cell profiling analyses suggest that distinct maturation and potentially physiological states underlie individual intermediate time points of hiPSC-CM differentiation. Hence, to determine if an intermediate time point of cardiac differentiation would contain distinct combinations of these populations, we next performed an independent single-cell RNA-seq analysis of an intermediate CM differentiation time-point (day 30) using the Fluidigm C1 system, which provides increased transcript coverage and the sensitivity to potentially resolve specific splice isoforms associated with cardiac maturity. Selecting 43 heavily sequenced hiPSC-CMs (4 million paired-end reads) with cardiac specific gene expression of cardiac troponin T Type 2 (*TNNT2)* and cardiac alpha actin (*ACTC1*) (Supplementary Fig. 1A-D), we were able to confirm the presence of distinct populations marked by *MYL2*, *MYH6*, *HOPX* and other markers identified from droplet-based sequencing analyses (see Materials and Methods). Unsupervised ICGS analysis of these cells combined with supervised analysis with guide-genes from Fig. 1b, we identified six distinct cell populations defined by the expression of splicing factor 1 (*SF1*), ISL LIM Homeobox 1 (*ISL1*), Jumonji AT Rich Interactive Domain 2 (*JARID2*), T-Box 5 (*TBX5*), Myosin Light Chain 2 (*MYL2*), or HOP Homeobox (*HOPX*) (Fig. 2c). While the transcription factor Nuclear Receptor Subfamily 2 (*NR2F2*) was highly expressed in the cell populations defined by *ISL1, JARID2* and *TBX5*, *MYL2* was expressed in a distinct subset of cells overlapping with the Hes-Related Family bHLH Transcription Factor and YRPW Motif 2 (*HEY2*). ICGS clusters using 10X single-cell RNA-seq from day 14 and day 45 cardiomyocytes also revealed the expression of these transcription factors (predominantly *HEY2* and *MYL2* expression) in later stage (day 45) cardiomyocytes compared to earlier time point cardiomyocytes (day 14) (Supplementary Fig. 1E). Pseudotemporal ordering of these cells with the software Monocle^13^ designated *SF1*-expressing cardiomyocytes as the “earliest” population and *HOPX* as the latest, suggesting that cardiomyocyte subpopulations underlie distinct cardiac maturation states (Supplementary Fig. 1F). These results are in agreement with our broader single-cell analyses of day 14 and 45 cardiomyocytes.

To determine if these transcription factors are likely to direct the expression of the associated gene-expression clusters, we next compiled gene expression signatures from several dozen transcription factor perturbation transcriptome experiments (e.g., knock-out, siRNA, and over-expression) with available supporting ChIP-seq data (Supplementary Data 1). Among the 20 transcription factors evaluated, significant enrichment in repressed *HEY2* targets were found in ISL1-expressing cells, whereas those repressed by *NR2F2* were enriched in the uncorrelated *HEY2/MYL2* population. Similar analyses of a dozen independent ontologies and curated gene sets found that the *NR2F2*-expressing cells were most highly enriched in atrial specific genes, whereas *HEY2/MYL2* were most highly enriched in ventricular morphogenesis genes (Fig. 2c, left panel). Hence, these data suggest that cardiac differentiation is defined by distinct transcriptional programs that can be associated with a more immature/atrial-like gene expression profile that transitions into a ventricular-like gene expression profile.

### Single-cell populations correlate with time points

Based on our bioinformatic predictions, we surmised that single-cell populations might underlie the cellular heterogeneity commonly observed in previous cardiac differentiation studies^4–6,14^. To test this hypothesis, we performed an in-depth bulk RNA-seq analysis of 13 time points during cardiac differentiation of iPSCs, spanning days 0 through 90 of differentiation, with three replicate differentiations (Supplementary Fig. 2A, Supplementary Data 2). Genes defining each stage of differentiation from this time course were obtained using the MarkerFinder algorithm within AltAnalyze, which selects genes with the greatest time point restricted expression pattern, ordering the genes within each sample set according to their relative specificity^15^. Gene Ontology enrichment analysis of the MarkerFinder-defined gene sets correspond to well-defined early mesoderm (day 1–3), cardiac progenitor specification (day 4–6), cardiac structural maturation (day 7–9), and contraction (day 14–90) markers. These expression changes were further characterized by established marker genes in mesoderm morphogenesis (*T, MIXL1*), early cardiac progenitors (*PDGFRA, MEF2C, BMP2, MKL2, PITX2, SNAI2, KDR*, and *MYOCD*), glycolysis (*GPI, HK1, HK2, PFKP*, and *ENO1*), sarcomeric assembly (*MYH6, CAPN3, NEB, EDN*, and *NKX2-5*), and cardiac contraction (*MYL2, HCN4, CASQ2, GPD1L, JAK2, DES, GJA5, PIK3R1, SCN5A, HEY2, FGF12*, and *DMD*) (Supplementary Fig. 2A, right panel). While PCA analysis of each time point of differentiation revealed a progression in gene expression signatures from hiPSCs to cardiomyocytes, maintained in culture for 90 days, significant heterogeneity among similar time point replicates were frequently observed (Supplementary Fig. 2B). To test whether these in vitro maturity differences were a result of underlying cell-state heterogeneity, we defined gene expression centroids from our single-cell time-course data for ICGS cell state-specific markers from MarkerFinder. Using the cell type classification algorithm LineageProfiler^12^ in combination with these marker gene profiles, we identified a gradual transition and overlap between predicted pluripotent stem cell, endoderm, mesoderm and cardiomyocyte subsets known to emerge at different time points from our droplet sequencing ICGS analysis (Supplementary Fig. 2C). Importantly, this analysis underscores the inherent differentiation-associated heterogeneity occurring among replicates collected at the same time points, possessing differential enrichment in early (day 5 and 14) versus late (day 90) in vitro progenitor populations. Hence, these results support the hypothesis that differences in cell type composition underlie the observed bulk gene expression changes observed in our time course data.

In addition to these global observations, our bulk RNA-seq time course data was used to evaluate the temporal specificity of single-cell maturation state predicted markers. The expression of *NR2F2* and *ISL1* is first observed at early differentiation time points (day 3–4), *TBX5* is expressed at intermediate time points (day 5–14), and *HEY2* and *HOPX* are expressed at late time points of differentiation (day 9, 14, 30, and 90) (Supplementary Fig. 2D). Examination of the predominant dynamically expressed transcriptional regulators in this time course highlights both the single cell population-specific genes along with established regulators (Supplementary Fig. 3).

### Global interactions of *NR2F2*, *TBX5*, and *HEY2* populations

**Fig. 3:**
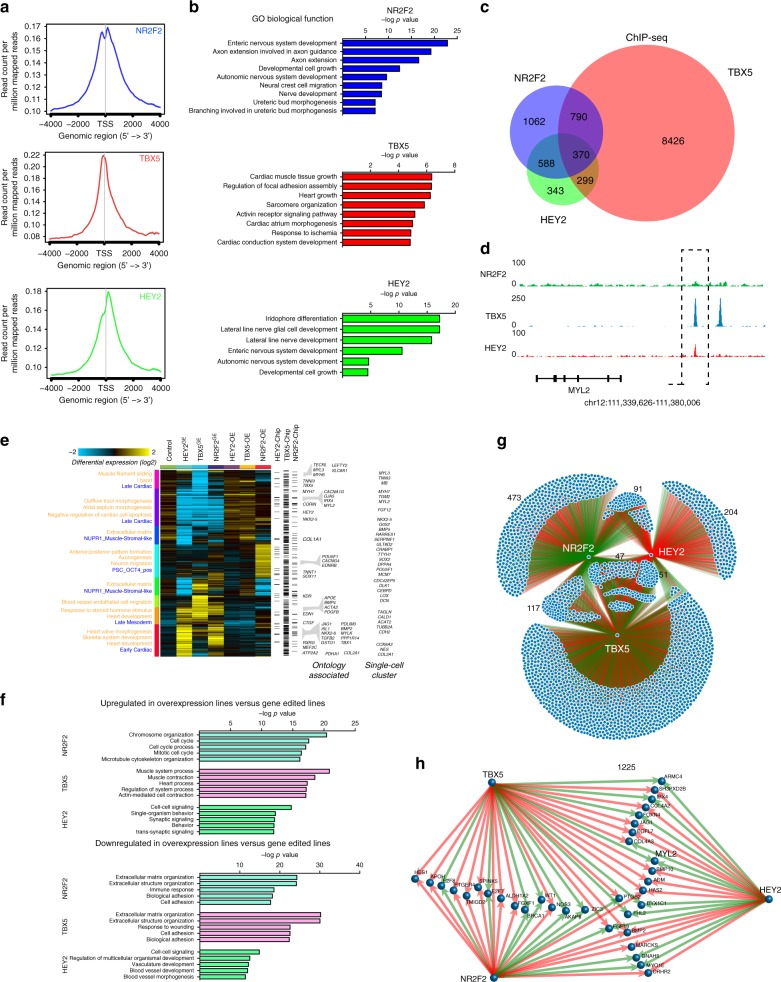
Transcriptome interactions between key transcription factors that regulate hiPSC-CM populations. ChIP-seq was performed on transcription factors (*NR2F2, TBX5*, and *HEY2*) enriched within three hiPSC-CM populations. **a** ChIP-seq peak density demonstrates enrichment of the ChIP-seq signal around the transcriptional start sites (TSS). **b** GO biological functions were graphed corresponding to peaks called within each ChIP-seq dataset (GREAT analysis, hypergeometric test, *p* < 0.05). *NR2F2* was highly enriched for terms within the nervous system, *TBX5* for cardiac tissue, and *HEY2* for neural and enteric terms. **c** Venn diagram demonstrates the number of unique and common genes called (using HOMER) within each ChIP-seq dataset. **d** Coverage of the reads demonstrating a peak near the *MYL*2 locus in the *TBX5* and *HEY2* ChIP-seq datasets, but not the *NR2F2* dataset. **e** RNA-seq was performed on cardiomyocytes from each gene-edited line [*NR2F2*^GE^ (*NR2F2*^GE1^ and *NR2F2*^GE2^ lines), *HEY2*^GE^ (*HEY2*^GE1^ and *HEY2*^GE2^), and *TBX5*^GE^ (*TBX5*^GE1^ and *TBX5*^GE2^)], as well as cardiomyocytes over expressing *NR2F2* (NR2F2^OE^), *TBX5* (TBX5^OE^) and *HEY2* (HEY2^OE^). Biological terms associated with genes significantly different (GO-Elite algorithm AltAnalyze) between these lines were annotated (left panel–blue text), as well as the corresponding ChIP-seq peaks identified (right panel). Gene sets associated with each of the single-cell populations from Fig. 1b are highlighted in orange. Genes associated with the enriched GO terms and single-cell populations are displayed to the right of the heatmap. **f** GO biological analysis was performed on significant genes upregulated or downregulated in hiPSC-CMs overexpressing *NR2F2, TBX5*, or *HEY2* with corresponding *NR2F2, TBX5*, or *HEY2* gene-edited lines. **g** Interactions among genes regulated by *NR2F2, TBX5*, and *HEY2* were visualized by combining the significant genes called within each *NR2F2*^GE1^*, TBX5*^GE1^, and *HEY2*^GE1^ with genes called within each ChIP-seq dataset. Nodes (blue circles) represent individual genes and the links represent the genes that interact with each transcription factor. Red indicates an upregulation and green represents a downregulation observed in the RNA-seq of each gene-edited line. **h** ChIP-seq/RNA-seq interactions using a selected set of cardiac-specific genes. MYL2 is highlighted to be positively regulated by *TBX5* and *HEY2* expression

Taken together, our single-cell gene and splicing analyses support a model in which distinct cell populations associated with opposing transcriptional regulators mediated cardiac maturation. To understand the broader transcriptional regulatory network associated with these population-specific factors, we next performed ChIP-seq using previously validated antibodies for *NR2F2, TBX5*, and *HEY2*. In all three ChIP-seq analyses, known cis-regulatory binding sites (CisBP in HOMER) corresponding to each respective transcription factor were enriched in proximity to the transcriptional start sites (TSS) of target genes (Fig. 3a). Pathway enrichment analysis of the associated ChIP-seq peaks^16^ identified an enrichment in neuronal encoding genes for *NR2F2*, cardiac-specifying factors for *TBX5*, and neural-related and enteric-related genes for *HEY2* (Fig. 3b). Quantification of the amount of nearest called genes to each ChIP-seq peak dataset identified abundant genes called within the *TBX5* dataset and revealed a potential synergistic or antagonistic target regulation with *NR2F2* and *HEY2* transcription factors (Fig. 3c). As predicted from our single-cell analysis, common ChIP-seq peak regions were shared between different combinations of each transcription factor and associated with the regulation of cardiac differentiation-regulated genes, such as the ventricular marker *MYL2* (Fig. 3d).

To assess whether the identified ChIP-seq peaks could activate or repress the nearest called target genes, we next sought to identify how the transcriptome profile of cardiomyocytes changes when the expression of *NR2F2*, *TBX5*, and *HEY2* is perturbed. To disrupt the function of each transcription factor, we used clustered regularly-interspaced short palindromic repeats (CRISPR) technology to delete exonic regions that are common in multiple transcript variants from *NR2F2*, *TBX5*, or *HEY2*. Using a guide RNA specific for *NR2F2*, we created an isogenic hiPSC line harboring a large 89 bp deletion in exon 2 of *NR2F2* from chr15:96334128-96334216 (*NR2F2*^GE1^). Using a guide RNA specific for *HEY2*, we created an isogenic hiPSC line harboring a 15 bp deletion in exon 2 from chr6:125751839-125751854 (*HEY2*^GE1^). In addition, we included a hiPSC line previously created by transcription activator-like effector nucleases (TALEN) technology by our group which harbors a 29 bp deletion within exon 2 of *TBX5* from chr12:114403882-114403910 (*TBX5*^GE1^)^17^ (Supplementary Fig. 4A, B). Furthermore, to ensure all transcript variants were impaired, we generated three lines harboring additional large genomic deletions (whole exon deletions) by using guide RNAs flanking exons within each transcription factor, annotated as GE2 (Supplementary Fig. 4C). The *NR2F2*^GE2^ line was a heterozygous deletion at chr15:96,880,430-96,881,090. The *TBX5*^GE2^ line contained a homozygous 9.7 kb deletion at chr12:114,823,085-114,832,791 and the *HEY2*^GE2^ line contained a homozygous 371 bp deletion at chr6:126,075,494-126,075,865. To assess the impact that disruption plays on each transcription factor in cardiogenesis, we differentiated each line and performed RNA-seq. The differentially expressed and opposing genes within the *NR2F2*^GE^ and *HEY2*^GE^ lines corresponded to prior established targets for both respective factors (Fisher-Exact enrichment *p* < 0.01), validating the use of this approach (Supplementary Fig. 4D)^18,19^.

To verify the role of each transcription factor in cardiomyocytes, we next overexpressed *NR2F2* (*NR2F2*^OE^), *TBX5* (*TBX5*^OE^), and *HEY2* (*HEY2*^OE^) in day 30 hiPSC-CMs using lentivirus and performed RNA-seq. To aid in the analyses of the associated transcriptomic data, we compared the expression profiles of these genes relative to control differentiations at the level of known biological pathways and our single-cell informed population subsets, combining RNA-seq from gene-edited lines for each transcription factor (Fig. 3e, f). These analyses implicate distinct transcriptional regulators in converging and/or opposing in vitro maturation pathways. Specifically, *NR2F2*^GE^ resulted in enhancement of *NUPR1* positive muscle-stromal-like cell associated genes which emerged at 45 days of differentiation (Fig. 1b–d), whereas *HEY2*^GE^ or *NR2F2*^OE^ blunted this cell-type specific signature, in alignment with our predicted opposing role of these factors. In addition, *NR2F2*^OE^ resulted in an enrichment in pluripotent stem cell (*SOX2*, *POU5F1*) and neuronal associated gene expression (*GPM6A, NEFM, NRCAM*), while reducing expression of early (*COX6A2, SMTNL2*) and late (*MYH6, TNNI3*) cardiomyocyte associated genes. The predicted differentiation skewing observed in *NR2F2*^OE^ is consistent with this factor’s established role in regulation of Notch signaling during differentiation^20,21^. Consistent with our initial predictions, genes upregulated by *HEY2*^GE^ and downregulated by *HEY2*^OE^ include *NR2F2*, the Notch signaling genes (*JAG1* and *DLL3*), regulators of cardiac contractility (*MYL3, COX6A1*), neurogenesis (*INA, BASP1, NEFL*), and human atria enriched genes (*NR2F2, MYLK*, *RBP1*), while genes with the inverse signature included those enriched in heart ventricles (*PDLIM1, VTN, LPL*) and cardiac hypertrophy (*MYH7, CORIN, TCAP*). Surprisingly, genes displaying reciprocal upregulation by *NR2F2*^GE^ and downregulation by *NR2F2*^OE^ included *TBX5* and atrial enriched genes (*MYH6, KCNK3, ACTA2*) in addition to TGF-beta signaling (*LEFTY2, BMP4, GDF6, CDKN2B*) and elastic fiber formation (*MFAP2, FBLN1, VTN*), whereas the inverse pattern (in *NR2F2*^OE^) included muscle relaxation (*SLC8A1*, *CHGA*, *ATP2A2*) and neurogenesis (*MYEF2, BMP2, NEFM*) genes.

To further delineate how each transcription factor influences the cardiomyocyte transcriptome, we next performed a joint network analysis of our ChIP-seq and RNA-seq data. To visualize this, we plotted each gene as a node and color coded the linked interacting nodes using lines to demonstrate a repressive (red) or active (green) interaction between each transcription factor and the genes that were found to have changed (greater than 1.5-fold change) within the gene edited lines (Fig. 3g). As previously reported^21-23^, *HEY2* interactions are predominately repressive and *TBX5* regulates a substantial number of cardiac genes. In addition, we also identified multiple genes that were co-regulated by *NR2F2*, *TBX5*, and *HEY2*, and plotted a select set of the cardiac genes identified (Fig. 3h). Given that *NR2F2* is expressed at an earlier time point than *TBX5* and *HEY2*, the later expression of *HEY2* and *TBX5* likely contributes to the upregulation of gene expression at later stages of differentiation. Specifically, later expression of *HEY2* and TBX5 was shown by ChIP-seq and RNA-seq to positively regulate the expression of the ventricular marker *MYL2*.

### Ventricular and atrial marker expression in hiPSC-CMs

To further assess how the atrial-like and ventricular-like identities characterize hiPSC-CMs, we sought to analyze how genes enriched in human adult atria versus ventricles change during hiPSC-CM differentiation. We found that the expression of *MYL2* (MLC2V) is enriched within the right and left ventricle (RV, LV)^22,23^, whereas *MYL7* (MLC2A) is enriched within the right and left atrium (RA, LA) (Supplementary Fig. 5A)^24,25^. Analysis of gene enrichment over time revealed that ventricular markers (*IRX4* and *MYL2*) appeared only at later time points of differentiation (Supplementary Fig. 2), *MYL2* expression was highly correlated with the *HEY2*-expressing subpopulation, and mRNA expression of *MYL2* was lower in *TBX5*^GE^ and *HEY2*^GE^ lines. Since ventricular markers could only be observed at later time points in differentiation, we sought to determine how the atrial versus ventricular gene expression signature develops over time.

As the complete RNA-seq transcriptome of normal (un-diseased) human atria and ventricular heart tissue has not been reported, we next performed RNA-seq on atrial and ventricular tissue from a healthy heart that had been destined for a heart transplant, but was rejected due to a donor mismatch. By plotting the percentage of adult heart genes expressed within hiPSC-CMs over time, we observed a progressive increase in the number of adult heart genes expressed during prolonged culture (Supplementary Fig. 5B). To investigate whether ventricular markers are enriched in cardiomyocytes at later time points, we assessed the top 100 atrial and ventricular enriched genes (i.e., measuring the greatest fold-change with empirical Bayes moderated unpaired *t*-test assuming unequal variance with *p* < 0.05) (Supplementary Fig. 5C) and graphed the percentage of atrial versus ventricular enriched genes expressed throughout cardiac differentiation (Supplementary Fig. 5D). At day 5 of differentiation, a similar percentage of atrial-enriched and ventricular-enriched genes could be detected. However, at day 6 in culture, a higher percentage of ventricular enriched genes could be observed and an increase in ventricular-enriched genes were detected throughout differentiation.

Given that our single-cell RNA-seq of the wildtype and genome-edited lines suggested that *NR2F2, TBX5*, and *HEY2* can regulate atrial-like and ventricular-like signatures, we next quantified the expression of these transcription factors within the adult heart (Supplementary Fig. 5E). RNA-seq of the human atria confirmed that *NR2F2* and *TBX5* are specifically enriched within the atria, and *HEY2* is highly enriched within the ventricle. Similarly, immunofluorescent labeling of MLC2V and MLC2A (Supplementary Fig. 5A) also verified differential expression of these markers in the heart. RNA-seq quantification demonstrated that *MYL2* is highly expressed within ventricular tissue, while *MYL7* is enriched within atrial tissue (Supplementary Fig. 5F). Furthermore, RNA-seq coverage of *MYL2* and *MYL7* in differentiating hiPSC-CMs reveals that *MYL2* is only observed at later differentiation time points (e.g. day 30 and day 90) (Supplementary Fig. 5G).

### Single-cell protein quantification using CyTOF

Our transcription factor interaction model revealed that late expression of *TBX5* and *HEY2* regulate the mRNA level of *MYL2*. To validate this finding at the protein level, we analyzed the expression of MLC2V using time-of-flight mass cytometry (CyTOF) analysis on a separate unbiased hESC line at three time points in differentiation (day 8, day 18, and day 30) (Supplementary Fig. 6). hESC-CMs (HES3 line) were stained with a panel of 37 elemental isotope-conjugated antibodies and measured with mass cytometry (Supplementary Fig. 6B). To visualize and interpret the high-dimensional dataset generated, we applied the t-SNE algorithm based on seven cardiac markers preselected for the dataset, in which individual cells in the high-dimensional space were projected onto a two-dimensional map but their neighboring relationship was preserved. t-SNE segregated cells into spatially distinct regions based on the markers expressed, and the relative position of cells from each time point on t-SNE map strongly correlated with the temporal order of differentiation (Supplementary Fig. 6C). Similar to the mRNA expression of *MYL2* and *MYL7* throughout cardiac differentiation, MLC2A was highly expressed in the majority of cells at day 8, day 18, and day 30, whereas the expression of MLC2V could only be observed at day 30 (Supplementary Fig. 6D). Interestingly, the match between the continuum of marker expression and the established differentiation status on t-SNE map similarly suggests the progression MLC2A + cells to MLC2V + cells.

### *NR2F2, TBX5*, and *HEY2* regulate the cardiac action potential

**Fig. 4:**
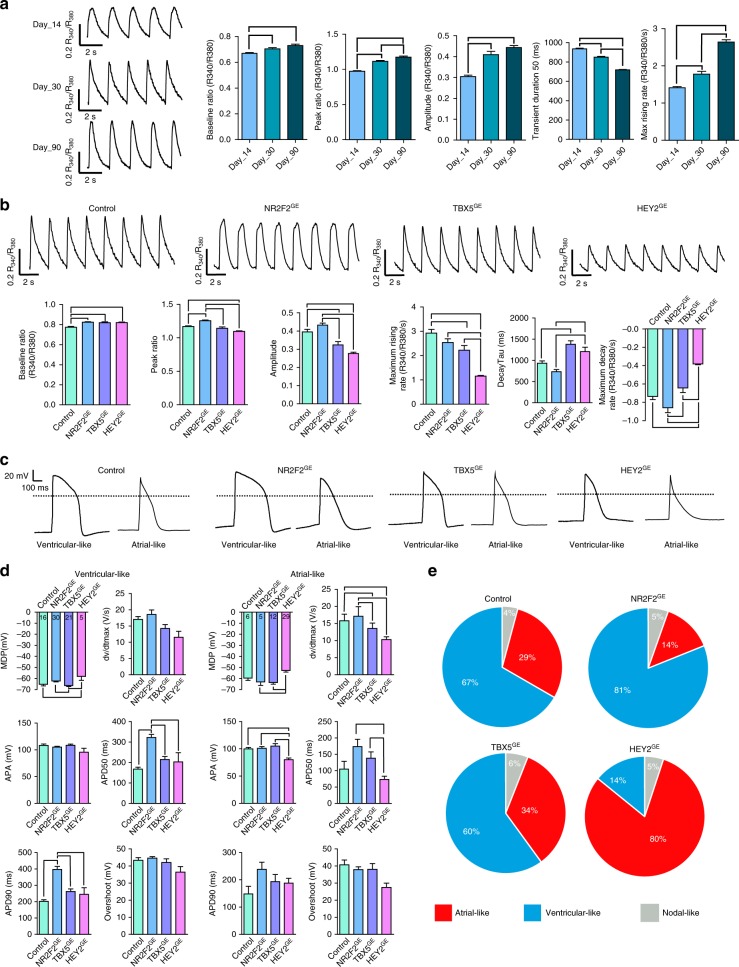
*NR2F2, TBX5*, and *HEY2* influence the electrophysiological properties of hiPSC-CM lines. **a** Representative traces of calcium imaging on hiPSC-CMs cultured for 14, 30, or 90 days in culture; over time, mature hiPSC-CMs presented a higher baseline ratio, peak ratio amplitude, maximum rising rate and lower transient duration 50. **b** Calcium imaging was performed on control (day 30-40) and each gene-edited line (day 30–40) to assess the calcium handling ability when *NR2F2*, *TBX5*, and *HEY2* were disrupted. In comparison to control hiPSC-CMs, *HEY2* lines displayed a significantly lower maximum rising rate and amplitude. **c** Representative patch clamp electrophysiological recordings for each gene-edited line. **d** Action potential parameters for 22 control, 35 *NR2F2*^GE^, 36 *TBX5*^GE^, and 34 *HEY2*^GE^ hiPSC-CMs at day 30-40 were quantified for either ventricular-like or atrial-like action potentials. Significantly lower maximum rising rate (dv/dt_max_), maximum diastolic potential (MDP), and action potential amplitude (APA) values were observed in the *HEY2*^GE^ line when compared to control lines. Increase in action potential amplitude (APD) 50 and APD90 was observed in the ventricular-like *NR2F2*^GE^ line. **e** The proportions of atrial-like, ventricular-like, and nodal-like action potentials were quantified within each gene-edited line. Slightly more ventricular-like action potentials were found in the *NR2F2*^GE^ line, whereas a large proportion of atrial-like action potentials were observed in the *HEY2*^GE^ line. Lines between bars represent *p* < 0.05 between groups using a one-way ANOVA

Pathway analysis performed using RNA-seq data from our gene-edited lines revealed that genes involved in calcium signaling were differentially expressed among the *NR2F2*^GE^, *TBX5*^GE^, and *HEY2*^GE^ lines. Given that *NR2F2*, *TBX5*, and *HEY2* were differentially expressed throughout different days in culture, we next sought to determine how calcium signaling changes in control hiPSC-CMs during prolonged culture (Fig. 4a). Calcium imaging (using the calcium indicator dye Fura-2 AM) performed on day 14, day 30, and day 90 revealed a significant increase in baseline ratio, peak ratio, amplitude, and maximum rising rate with a decrease in transient duration over time. Next, we assessed whether calcium signaling was affected in our day 30 *NR2F2*^GE^, *TBX5*^GE^, and *HEY2*^GE^ gene edited lines (GE represents data from both GE1 and GE2 lines) (Fig. 4b). Interestingly, our ratiometric calcium imaging with Fura-2 AM revealed a significant decrease in the transient amplitude, immature calcium dynamics (shown as decreased maximum rising/decay rate), and slower calcium recycling rate (indicated by prolonged decay Tau) in *TBX5*^GE^ and *HEY2*^GE^ lines when compared to control day 30 hiPSC-CMs. All of these properties suggested that *TBX5*^GE^ and *HEY2*^GE^ lines had a less mature phenotype. Finally, to determine whether *NR2F2*, *TBX5*, and *HEY2* are involved in the heterogeneous atrial- and ventricular-like action potential as previously reported^4–6^, we performed patch clamp analysis on control, *NR2F2*^GE^, *TBX5*^GE^, and *HEY2*^GE^ lines (Fig. 4c, d). Consistent with more immature and atrial-like phenotype of cardiomyocytes, action potentials within the *HEY2*^GE^ group had a significantly more depolarized minimum diastolic potential (MDP), lower upstroke velocity, lower dv/dt_max_, and lower action potential amplitude (APA) compared to control cells (one-way ANOVA, *p* < 0.05). In addition, *NR2F2*^GE^ hiPSC-CMs displayed significantly higher APD50 and APD90 values compared to control hiPSC-CMs. Differences within AP parameters (ADP90, ADP50, and overshoot) therefore suggest that action potential parameters are significantly influenced by *NR2F2* and *HEY2*. During days 30-40, the clear majority (73%) of control, *NR2F2*^GE^, and *TBX5*^GE^ hiPSC-CMs displayed ventricular-like action potentials whereas the majority of *HEY2*^GE^ action potentials exhibited atrial-like action potential morphology. These findings suggest that *HEY2* is an important regulator of the ventricular-like action potential identity (Fig. 4e).

## Discussion

The heart has a limited regenerative capacity, and finding a suitable cardiomyocyte population to replace the cardiomyocytes lost after injury would be extremely useful. Researchers now can use hiPSC-CMs to repair the damaged heart, to model patient-specific heart diseases, and to test drug responses; however, a more thorough understanding of hiPSC-CMs is still needed. Recent studies indicate that hiPSC-CMs represent a heterogeneous population displaying atrial-like, ventricular-like, or nodal-like action potentials^4–6^, and numerous studies suggest that these cells are immature relative to their adult counterparts^3,26–28^. Given this reported heterogeneity, we sought to identify the genetic signatures of these distinct populations, as well as provide mechanistic insight into how each population arose. By using single-cell RNA-seq, we identified subpopulations of cardiomyocytes characterized by distinct underlying gene signatures, subpopulation specific splicing events, and the enrichment of specific cardiac transcription factors regulating these subpopulations. Downstream analysis of three transcription factors revealed that these subpopulations are related to different stages of cardiac differentiation, as well as correlated with atrial-like or ventricular-like gene signatures.

Single-cell RNA-seq on hiPSC-CMs from the same day after differentiation (day 30) identified multiple subpopulations enriched for *TBX5, NR2F2, HEY2, ISL1*, *JARID2*, or *HOPX* transcription factors. By assessing the expression of these transcription factors during differentiation, we observed that the temporal expression pattern is correlated with unique subpopulations. In conjunction with single-cell pseudotemporal ordering and bulk RNA-seq comparison analyses, these data suggest a model by which hiPSC-CMs from different days in culture represent a pooled profile of cardiomyocytes from different differentiation stages and even distinct mesoderm-derived cell populations. This is particularly evident in the cardiomyocyte subpopulation expressing *ISL1*. Because *ISL1* is a well-described cardiomyocyte progenitor marker^29,30^, the temporal expression of this transcription factor during cardiomyocyte differentiation supports the idea that cardiomyocyte subpopulations represent different stages of differentiation.

Our global single-cell analyses highlight transcriptional heterogeneity associated with reported atrial (*NR2F2/TBX5*) versus ventricular (*HEY2/MYL2*) enriched gene expression change. Given that an atrial enriched gene profile surfaces prior to the expression of ventricular markers (*IRX4* and *MYL2*), we surmised that in using this small molecule differentiation protocol, ventricular destined cardiomyocytes may progress from an immature/atrial-like (early stage) to a ventricular-like gene expression profile. In this case, transcriptional regulators associated with these distinct cardiomyocyte cell states would govern the switch from an atrial- to ventricular-like state. Integrating our single-cell RNA-seq results with patch clamp and gene expression profiling of *NR2F2* and *HEY2* genome-edited lines, provides strong evidence for the distinct regulatory role of *NR2F2* versus *HEY2* in promoting atrial-like versus ventricular-like functional cell states, respectively. The role that *NR2F2* plays in regulating atrial characteristics is supported by studies using retinoic acid to increase the expression of *NR2F2* and to generate atrial-like hiPSC-CMs^31,32^.

The role that *NR2F2* and *HEY2* play in regulating an atrial-like or ventricular-like gene expression profile has also been reported in mice. By ablating Nr2f2 in the mouse myocardium, ventricularization of the atrium was observed. In addition, ectopic expression of Nr2f2 in the ventricle myocardium atrialized the ventricular myocardium^33^. Hey2-deficient mice died within the first week after birth due to cardiovascular defects (ventricular septal defects, AV valve irregularities, and cardiac hypertrophy), but mice defective in Hey genes produced ectopic activation of atrial genes within the ventricular myocardium. Furthermore, forced expression of Hey2 (Hrt2) in atrial cardiomyocytes was found to suppress atrial specific genes. Here, we further show that *NR2F2* and *HEY2* expression are mutually exclusive, with *NR2F2* displaying robust expression in the human atrium and *HEY2* in the human cardiac ventricle. Given that *HEY2* belongs to the notch signaling pathway and that significant regulation of key Notch signaling proteins are found with the perturbations presented here, it is possible that notch signaling may be regulating the atrial versus ventricular specific gene signatures within different heart chambers.

To model and understand cardiac disease, it is necessary to identify the molecular and cellular sources of heterogeneity in differentiating hiPSC-CMs. Here we combined our RNA-seq data with transcription factor targeting analysis (ChIP-seq) to derive a co-regulatory and counter-acting system of transcription factor interactions centered on *NR2F2, TBX5*, and *HEY2*. Interestingly, we observed common targets between these transcription factors, suggesting the involvement of multiple transcription factors in regulating the expression of cardiac genes. These data provide strong complementary evidence that *NR2F2* regulates genes promoting an earlier atrial-like state, whereas the induction of *HEY2* promotes a more mature (i.e. later differentiation) ventricular-like gene expression profile. This model is supported by our RNA-seq data, which found genes enriched within the adult cardiac ventricles (*MYL2, NAV1, SLC5A1, ACTA1*) that were higher in the *HEY2* overexpressing cardiomyocytes, with subsequent decreased expression of these genes in *HEY2* gene edited lines. Hence, aberrant signaling through transcription factors enriched within a given heart chamber may be responsible for regional pathology observed in some heart diseases. This possibility is also supported by the positive correlation observed between cardiac defects involved in Holt–Oram syndrome (caused by *TBX5* mutations) with the spatial expression of Tbx5^34^.

We believe that these analyses present a powerful strategy for gaining novel integrated cellular, molecular and physiological insights into differentiation-associated heterogeneity. This work represents only the beginning of such investigations, given that additional downstream studies will be needed to fully characterize the several additional novel cell states found to emerge through hiPSC-CM differentiation (Fig. 1b). Understanding which factors regulate these cell fate decisions is likely to improve our ability to generate enriched cardiomyocyte subtypes of differing maturity and functionality. In the future, we believe that this type of integrative single-cell analytical approach is likely to shed light on additional complex cellular systems, beyond cardiac maturation.

## Methods

### Generation of human induced pluripotent stem cells

A skin punch biopsy was performed and dermal fibroblasts were acquired from this biopsy under informed consent as outlined in and approved by Stanford’s Institutional Review Board (IRB) protocols. Fibroblasts were grown in DMEM supplemented with 10% fetal bovine serum (FBS) and 1% pen/strep on gelatin-coated flasks. Fibroblasts were passaged using trypsin and 1 × 10^6^ fibroblasts were electroporated using 10 µg of reprogramming episomal vectors^35^ using the Neon® Transfection System. Fibroblasts were plated onto Matrigel™-coated 10 cm culture wells in DMEM supplemented with 10% FBS, 1% pen/strep, and hydrocortisone. Fibroblasts were then grown using Essential 7 (E7) media [Essential 6 (Invitrogen) with FGF2 (50 µg/L)] and sodium butyrate (0.2 mM NaB) (B5887 diluted in DMSO, Sigma) for thirteen days and then switched to Essential 8 (E8) media (Invitrogen) until hiPSC colonies were formed. hiPSC colonies were picked and expanded until passage 10 before being used in experiments outlined below. hiPSCs were tested to be mycoplasma negative using the Mycoalert Mycoplasma testing kits (LT07-318, Lonza).

### Cardiomyocyte differentiation

Cardiomyocytes were differentiated using a monolayer method as previously described^11^ with minor modification. hiPSCs (passage 24-35) were seeded at 1.2 × 10^5^ per well in Matrigel™-coated 6 cm culture wells and grown for four days prior to starting hiPSC-CM differentiation. To initiate differentiation, B27 without insulin (A1895601, Life Technologies) in RPMI supplemented with 6 µM CHIR-99021 (CT99021, Selleckchem) was added to the hiPSCs for two days. CHIR was removed from the cultures and cells were incubated for one day in RPMI supplemented with B27 without insulin. Cultures were then treated with 5 µM IWR-1 (I0161, Sigma) in RPMI supplemented with B27 without insulin for two days and incubated for two days with RPMI containing B27 without insulin. For two days, cultures were maintained in RPMI with B27 with insulin (17504-044, Life Technologies) and glucose starved for three days (using RPMI minus glucose). After glucose starvation, hiPSC-CMs were maintained in RPMI with B27. Cardiomyocyte differentiation was repeated three additional times and the cells were then processed for RNA-seq.

### Overexpression of *NR2F2*, *TBX5*, and *HEY2* in hiPSC-CMs

To overexpress *NR2F2*, *TBX5*, and *HEY2* in hiPSC-CMs, lentiviral particles were purchased for mGFP-tagged *NR2F2* (Cat# RC206753L2V), *TBX5* (Cat# RC216520L2V), *HEY2* (Cat# RC202544L2V), and control *GFP* Lent-ORF particles (Cat# PS100071V5) from Origene. Two sets of day 30 hiPSC-CMs were infected with 1 or 2 MOI of each lentivirus and maintained in culture for 1 week. RNA from *NR2F2*, *TBX5*, *HEY2*, and *GFP* infected hiPSC-CMs was extracted and RNA-sequenced using Illumina’s HiSeq 4000 2 × 150 paired end sequencing (Novogene).

### RNA-sequencing

RNA-seq of cardiomyocyte differentiation (*N* = 3 separate differentiations) from hiPSCs was performed as follows: total RNA was isolated using the miRNeasy Micro Kit (Qiagen). 100 ng of RNA was converted to cDNA using the Ovation® RNA-Seq System V2 kit (NuGEN) as outlined in the manufacture’s protocol. cDNA was fragmented using the Covaris S2 to an average fragment size of 300 bp and the fragmented cDNA was purified using Agencourt AMPure XP beads. End repair, dA tailing, and adapter ligation was performed using the NEBNext DNA Library Prep Master Mix Set for Illumina (E6040L, NEB) with 500 ng sheared cDNA input. Size selection was performed on the Pippen Prep using 2% DNA gels (12-100-506, Sage Science) to capture 300–360 bp DNA fragments. Bar-coded RNA-seq libraries were pooled and hybridized to one lane of an Illumina HiSeq 2000 flow cell using 2 × 100 paired end reads on the HiSeq 2000.

Two hiPSC lines were obtained from the Stanford Cardiovascular Institute biobank (CVI0076, CVI0059). CVI0059 was processed for single cell RNA-seq at day 5, day 14, and day 45 of the cardiomyocyte differentiation protocol using the 10X Genomics single-cell RNA-seq v1 kit. CVI0076 was processed for single cell RNA-seq at day 0, day 5, day 14, and day 45 of the cardiomyocyte differentiation protocol using the 10X Genomics single-cell RNA-seq kit v2. Single-cell RNA-seq was performed as follows. Cells were trypsinized for 5 min and filtered through a 60 μm pore filter. Trypsin was removed by centrifugation and cells were washed three times with PBS + 0.04% BSA (Non Acetylated, Sigma B6917). Cells were submitted to the Stanford Functional Genomics facility for single-cell library preparation and sequencing. Briefly, Gel-Bead in Emulsions (GEMs) were generated using the 10X Chromium system (10X Genomics, Pleasanton, CA). Barcoded cDNA was extracted from the GEMs by Post-GEM RT-cleanup and amplified for 12 cycles. Amplified cDNA was sheared (target –200 BP, Covaris S2), and subjected to end-repair, poly A-tailing, adapter ligation, and 10X specific sample indexing as per manufacturer’s protocol. Libraries were quantified using Bioanalyzer (Agilent) and qPCR (KAPA) analysis. Libraries were sequenced on the NextSeq 500 (Illumina). Unsupervised cell population discovery analyses were performed with Seurat-CCA and the software ICGS available in AltAnalyze version 2.1.1 (http://www.altanalyze.org)^12^. For these analyses, only protein-coding genes were considered, applying a correlation cutoff of 0.3 and Euclidean column HOPACH clustering. Associated t-SNE visualizations were obtained in AltAnalyze using ICGS obtained dynamically regulated genes.

Single-cell hiPSC-CMs (day 30 after differentiation began) were captured using 10–17 µM C1 Single-Cell Auto Prep IFC for mRNA-Seq (Cat# 5760, Fluidigm). Single-cell RNA-seq libraries were further generated according to the manufacturer’s protocol using the SMARTer Ultra Low RNA Kit for the Fluidigm C1 (Clontech) and Nextera XT DNA Sample Preparation Kit (FC-131-1096, Illumina). Each captured cell was labeled using a live and dead staining kit (L-3224, Life technologies) and imaged using florescence microscopy in the capture site to ensure viable, single cells were captured at each site and that these cells did not represent doublets or dead cells. Cells were captured in 85 of the 96 capture sites, 19 of which were considered dead based on the florescent ratio of the dead versus live stain and 15 sites with one or more cells observed (aka doublets, 18%). The observed number of doublets for a single medium C1 chip is similar to the expected rate suggested by the manufacturer (~30% with a standard deviation of 10%). Doublets were defined as two or more cells that reside in either the wings of the capture site or that are stacked on top of each other in the capture site nest, as per the manufacturer recommendations. ERCC spike-ins were included for further evaluation of sample quality. For the 51 libraries corresponding to single-live cells, libraries were pooled and sequenced using Illumina’s HiSeq 2000 using 2 × 100 paired-end sequencing (Macrogen, South Korea). Filtered reads were aligned to the reference genome hg19 using STAR^36^. Using STAR BAM files, AltAnalyze was used to generate exon read counts for gene expression analysis and junction read counts for splicing analysis (see below). All retained single-cell libraries were required to have a minimum of 1 million uniquely aligning paired-end fragments and > 40% aligned fragments, based on STAR analysis. The retained libraries had an average of ~3 million aligned fragments. Analysis of these same libraries using the Kallisto and TopHat2 workflows produced similar estimated and percentage aligned fragment statistics^37,38^. This filtering excluded only two of the 51 libraries, both of which had less than ~6,000 aligned fragments, with all retained cells having over one million aligned reads. In addition, we applied a stringent filtering of the cells based on the number of exon-exon junctions expressed ( > 400,000 junctions reads/cell). To calculate RPKM values for each gene, AltAnalyze was run on the junction and exon BED files using default settings. To identify discrete cell states, unsupervised clustering was initially performed to define predominant populations (ICGS module of AltAnalyze, Pearson correlation coefficient > 0.4). Although this analysis identified three initial populations, we augmented these results using a supervised analysis of cardiac transcription factors from our 10X Genomics identified using the ICGS supervised correlation option. In agreement with our Fluidigim C1 microscopy analyses, no gene expression signatures with evident “doublet cell” profiles (more than one cell population signature) were discerned from this analysis. Furthermore, ERCC spike-in expression (ERCC92.fa, Kallisto TPM) ratios indicated single-cell transcriptome profiles were being assessed. Using the obtained cell populations from this supervised analysis, the MarkerFinder algorithm in AltAnalyze was run to identify additional genes with population-restricted expression profiles (Pearson correlation coefficient > 0.4).

RNA-seq libraries for control, *NR2F2*^GE1^, and *HEY2*^GE1^ hiPSC-CMs were created using the Ion AmpliSeq™ Transcriptome Human Gene Expression Kit (Life Technologies). Each library was adjusted to a 100 pM concentration and Ion Template preparation was performed using the automated Ion Chef system. Templates were loaded on the Ion PI™ Chip Kit v3 (Life Technologies) and sequencing was performed using the Ion Proton sequencing platform with the Ion PI™ Hi-Q™ Sequencing 200 Kit (Life Technologies). Quantification of gene expression differences was performed using the Ion Torrent Suite software (Life Technologies) with the hg19_AmpliSeq_Transcriptome_21K_v1 reference. Additional differentiations were performed on *NR2F2*^GE1^ (*N* = 2), *TBX5*^GE1^ (*N* = 2)*, HEY2*^GE1^ (*N* = 2)*, NR2F2*^GE2^ (*N* = 4), *TBX5*^GE2^ (*N* = 3), and *HEY2*^GE2^ (*N* = 2) lines and sequenced using Illumina’s HiSeq 4000 2 × 150 paired end sequencing (Novogene).

### ChIP-sequencing

Cardiomyocytes (day 20 of culture, *N* = 1) were crosslinked by 1% formaldehyde and sheared using tip sonicator (450D, Branson). A small portion of the crosslinked, sheared chromatin was saved as the input. Five micrograms of either anti-NR2F2 (61213, Active Motif), anti-HEY2 (10597-1-AP, Proteintech) or anti-TBX5 (SAB1411311, Sigma) was incubated with protein G Dynabeads (10003D, Invitrogen) for 12 h at 4°C and the remainder were incubated with the antibody conjugated Dynabeads. After overnight incubation at 4°C, the incubated beads were rinsed with sonication buffer (50 mM HEPES pH 7.9, 140 mM NaCl, 1 mM EDTA, 1% Triton X-100, 0.1% Na-deoxycholate, 0.1% SDS, 0.5 mM PMSF), high salt buffer (50 mM HEPES, pH 7.9, 500 mM NaCl, 1 mM EDTA, 1% Triton X-100, 0.1% Na-deoxycholate, 0.1% SDS, 0.5 mM PMSF), and LiCl buffer (20 mM Tris, pH 8.0, 1 mM EDTA, 250 mM LiCl, 0.5% NP-40, 0.5% Na-deoxycholate, 0.5 mM PMSF). The washed beads were incubated with elution buffer (50 mM Tris, pH 8.0, 1 mM EDTA, 1% SDS, 50 mM NaHCO_3_) for 1 h at 65°C and then de-crosslinked with 5 M NaCl overnight at 65°C. The immunoprecipitated DNA was treated with RNase A and Proteinase K, and purified by ChIP DNA clean and concentrator (D5205, Zymo Research). 10 ng was used as input DNA into the NEBNext® Ultra™ DNA Library Prep kit to add on sequencing adapters, and a fragment size of 420–580 bp was isolated using the Pippen Prep (Sage Science). Bowtie was used to map the raw sequencing reads to the reference hg19 genome and peak calling was performed using MACS v2.0.10^39^. To annotate peaks to the nearest transcriptional start site, Bedtools (Quinlan and Hall, 2010) and HOMER (http://homer.salk.edu/homer/) were used. Peak bed files were also used as input into GREAT to identify Biological Process Gene Ontology terms^16^. Lists of common ChIP-seq annotated genes and genes demonstrating a 2-fold change in expression between control versus each gene-edited line (RNA-seq) were generated to identify genes likely regulated by each transcription factor. Commonly called genes between ChIP-seq datasets and RNA-seq datasets were merged to generate a list of all transcription factor to gene interactions. Visualization of each interaction between all genes (transcription factor interaction maps) was generated with a modified code of the 3D Force layout using threejs (http://d3js.org/). Nodes represented individual genes, the links represented an interaction between two genes, and the color of the link indicated either an upregulation (green) or a downregulation (red) observed in the RNA-seq datasets.

### Gene editing cardiac transcription factors

*TBX5* gene-edited line was previously created and described^17^. Briefly, TALEN binding sites were designed using the TAL Effector Nucleotide Targeter 2.0 having a repeat array length of 15 repeat variable di-residue domains and a spacer length of 14–18 nucleotides^40^. *TBX5* TALEN was generated from a plasmid library through a five-piece subcloning ligation: three sequence-specific tetramer-recognition pieces, one trimer-recognition piece, and an expression vector backbone (pTAL) as previously described^41^. The forward construct (containing the *TBX5* start and reverse TALENs) were subcloned into the pTAL_GFP and pTAL_RFP backbones. hiPSCs seeded at 2 × 10^6^ cells were transfected with a pair of TALENs (1.0 μg of each TALEN) by nucleofection using the P3 Primary Cell Nucleofector Kit and program CM-150 per the manufacturer’s instructions on the Amaxa 4D Nucleofector system (Lonza). Double GFP + /RFP + cells were sorted by FACS (FACSAria II; BD Biosciences) and clonal selection was performed as described below.

CRISPR guide RNAs were designed using the Zhang lab’s online CRISPR design tool (http://crispr.mit.edu/). Guide regions were designed to represent exons shared by multiple transcript variants of each gene (*NR2F2* or *HEY2*) (Supplementary Fig. 4). Guides were cloned into the pSpCas9(BB)-2A-GFP vector (gift from Feng Zhang: #48138, Addgene) and 3 µg transfected into hiPSC cells using Lipofectamine® 3000 reagent. Two days after transfection, GFP expression was observed. GFP expressing hiPSCs were incubated for 5 min in Accutase to release hiPSCs from the culture plate. hiPSCs were passed through a 100 µm cell strainer and Accutase was removed by centrifugation at 300×*g* for 4 min. hiPSCs were resuspended in 250 µL of FACS wash buffer (1% BSA in PBS) and FACS was then performed using the FACSAria III cell sorter (BD Biosciences). Sorted cells were seeded at a density of 1000 cells per well of a 6-well culture plate and were clonally expanded for 7 days. To identify the genotype of each hiPSC clone, genomic DNA was isolated from each CRISPR targeted clone using the DNeasy Blood & Tissue Kit (Qiagen), and PCR was performed on the region spanning the CRISPR targeted site using PrimeSTAR® GXL DNA Polymerase (Clontech). Each PCR amplified region was Sanger-sequenced (Quintara Biosciences, San Francisco) to validate the targeted deletion. In addition, the PCR product was TOPO-cloned using a StrataClone Blunt PCR Cloning Kit (Agilent), and plasmids from six clones (per PCR product) was purified using QIAprep Spin Miniprep Kit (Qiagen) and sequenced using the T3 primer (Quintara Biosciences). Sequencing each clone identified a large 89 bp deletion in exon 2 of *NR2F2* from chr15:96334128-96334216 (*NR2F2*^GE^), as well as a 15 bp deletion in exon 2 of *HEY2* from chr6:125751839-125751854 (*HEY2*^GE^) (Supplementary Fig. 4B). *NR2F2*^GE2^, *TBX5*^GE2^, and *HEY2*^GE2^ lines were created using the background corresponding *NR2F2*^GE1^, *TBX5*^GE1^, and *HEY2*^GE1^ cell lines. Two CRISPR guide-RNAs targeting sequences upstream of each exon (NR2F2: CAAACTGCCCCAACCGGAGT and ATTTGCTCCAACTCCGGTTG, TBX5: GTTGGCGCCATTGGGCAACC and TCACATGTGGTTGGCGCCAT, HEY2: AGTTGGGATTGTCTAGTGAG and GTTGGGATTGTCTAGTGAGA), as well as two guide-RNAs targeting sequences downstream of each exon (NR2F2: CAAGTTGTTCTGACCGACAC and AGCTAGAGGTACATAGACAC, TBX5: CAAGGCGAATTTAGAGGGCG and GCGGGGAGCAGGGTTTTATC, HEY2: AATGGCAGGATTGAACTCGT and TAATGGCAGGATTGAACTCG) were used to create large deletions within each transcr-iption factor using the same protocol outlined above. Clones were picked, and sequence verified to reveal successful excision of each exon. PCR was also used to verify deletions (using the primers: NR2F2: NR2F2-FGATCGTGGACGCCATTAC NR2F2-R GTGCGTTTCCATCATCTTTG, TBX5: TBX5-F TTAGCACCAGCTTCCAATC TBX5-R GTTCTCTCTTCCTCTTTCCTTC, HEY2: HEY2-F TTTGCTGTGGTGATCTTAGG HEY2-R ATTACCTCTAACCTCCTGTTTATG) within each region targeted (Supplementary Fig. 4C).

### Immunocytochemistry and immunohistochemistry

Human heart atrial and ventricular tissue was acquired under Stanford IRB approval. A 0.5 cm^2^ piece of heart tissue was placed in 3.8% formalin for 5 h. After 5 h, samples were stored at 4°C in PBS and submitted to the Department of Comparative Medicine’s Histology Lab for paraffin embedding and tissue sectioning. 10 µM sections were fluorescently labeled as previously described^42^ using the antibodies ATTO^®^ 550 conjugated anti-MLC2V (1:200, *MYL2*: 310-111AT2, Synaptic Systems) and ATTO^®^ 647 N conjugated anti-MLC2A (1:200, *MYL7*: 310-011AT1, Synaptic Systems). Sections were also stained using DAPI (1:1000) and imaged using a confocal microscope (Zeiss LSM-510) in the Neuroscience Microscopy Service Facility (Stanford University).

hiPSC-CMs were trypsinized after cardiac differentiation (day 9) and seeded onto Matrigel®-coated 35 mm coverslips. hiPSC-CMs were maintained in RMPI/B27 media until day 14, day 30, or day 90 after differentiation and fixed using ice-cold 80% methanol/20% acetone at 4 °C for 20 min. Cells were rinsed with PBS for 5 min (repeated three times) and blocked using 3% Bovine Serum Albumin (BSA; BP1600-100, Fischer BioReagents). Cells were labeled with the same antibodies and concentrations used to label from the heart tissue immunohistochemistry section above.

### Single-cell CyTOF analysis of hESC-CM differentiation

Human ESC-CMs (HES3, WiCell)^43^ were collected at the indicated time points, labeled with IdU to assess cell proliferation, and with cisplatin for live–dead cell discrimination as previously described^44,45^ (Supplementary Fig. 5A). Cells were then treated with 1 × TrypLE (Invitrogen) for 4 min at 37°C, dissociated into single-cell suspension by trituration, filtered through a 40 um filter and fixed with 1.6% paraformaldehyde at room temperature for 10 min followed by two washes with Cell Staining Medium (CSM, PBS containing 0.5% BSA). Formaldehyde-fixed cell samples from different time points were individually mass tag barcoded as previously described^46^. Individual samples were then pooled and incubated with metal-conjugated antibodies against surface markers for 1 h, washed once with CSM, permeabilized with methanol on ice for 15 min, washed twice with CSM and then incubated with metal-conjugated antibodies against intracellular molecules for 1 h. Cells were washed once with CSM, and incubated at room temperature for 20 min with an iridium-containing DNA intercalator (Fluidigm) in PBS containing 1.6% paraformaldehyde. After intercalation/fixation, the cell samples were washed once with CSM and twice with water before measurement on a CyTOF mass cytometer (Fluidigm). Normalization for detector sensitivity was performed as previously described^47^. After measurement and normalization, the individual FCS files were analyzed by first gating out doublets, debris and dead cells based on cell length, DNA content, and cisplatin staining. viSNE maps were generated with software tools available at www.cytobank.org using the indicated markers to perform clustering^48^.

### Ratiometric Ca^2+^ transient imaging

Differentiated beating hiPSC-CMs were dissociated by Accutase and replated in Matrigel (BD Bioscience) pre-coated 25 mm round cover glass (Warner Scientific Inc.). Cells were recovered for 3-4 days. For Fura-2 AM imaging, cells were loaded with 10 µM Fura-2 AM (stock solution of Fura-2 AM was pre-dissolved in 20% Pluronic F-127 solution in DMSO) in Tyrode’s solution (140 mM NaCl, 1 mM MgCl_2_, 5.4 mM KCl, 1.8 mM CaCl_2_, 10 mM glucose, and 10 mM Hepes pH = 7.4 with NaOH at RT) for 30 min at room temperature. To pace cells, we used a custom-made LabView script to generate pulse waveforms with a stimulus isolation unit (Warner Scientific Inc. SIU-102) that generates pulse signals to stimulate hiPSC-CMs in the imaging chamber (Warner Scientific Inc. RC-21BRFS). Cell samples were activated with Lamda-4 light source (Sutter) with fast switching between 340 and 380 nm wavelength, and the emission signals were recorded with high-frame rate EMCCD (Andor iXon Ultra897) that was connected to a reverse fluorescence microscope (Nikon. Ti-S) with a 40X oil immersed objective (CFI Plan Fluor 40 × , NA 0.75). Signaling was captured in fast frame rate video mode (512 × 512 frame) at a speed of 50 fps. For data analysis, a custom-made script with Mat-lab was used. Transient amplitude was expressed as R340/R380.

### Electrophysiological recordings

The whole-cell patch clamp recordings were performed using the standard patch clamp technique as previously described^14^. Briefly, contracting monolayers of control and knockouts of *NR2F2*, *HEY2*, and *TBX5* hiPSC-CMs from days 30–40 were enzymatically dispersed into single cells using Accutase, and attached to Matrigel-coated glass coverslips (Warner Instruments) in RPMI medium supplemented with B27. After 72 h of incubation, action potentials were recorded from single cells in the current clamp mode at 36–37 °C, using an EPC-10 patch-clamp amplifier (HEKA, Lambrecht, Germany) attached to a RC-26C recording chamber (Warner Instruments) and mounted onto the stage of an inverted microscope (Nikon, Tokyo, Japan). The single cells were paced at constant pacing rate of 1 Hz using a 5–20 ms depolarizing current injections of 150–550 pA. The glass pipettes were fabricated from standard wall borosilicate glass capillary tubes (BF 100-50-10, Sutter Instruments) using a P-97 Sutter micropipette puller, and fire polished with a microforge (Narishige, MF830, Japan) to generate electrodes with tip resistances from 2-4 MΩ. Attached cells on coverslips were continuously perfused with the extracellular solution containing (in mM) 150 NaC1, 5.4 KCl, 1.8 CaCl_2_, 1.0 MgCl_2_, 15 HEPES, 15 glucose, and 1 sodium pyruvate (pH adjusted to 7.4 with NaOH). The intracellular solution contained (in mM): 120 KCl, 1 MgCl_2_, 10 HEPES, 3 Mg-ATP, and 10 EGTA (pH adjusted to 7.2 with KOH). Data were acquired using PatchMaster software (HEKA), digitized at 1.0 kHz, and analyzed using FitMaster (HEKA, Germany), Igor Pro (Wave Metrics) and Origin 2016 (OriginLab, Northampton, MA). All data were expressed as mean ± S.E.M., and statistical significance was determined using one-way ANOVA, with the significance level set at *P* < 0.05. We used the APD_90_/APD_50_ ratio, which describes the shape of the repolarization curve, to determine the specific subtype of each hiPSC-CM^49^. hiPSC-CMs with low APD_90_/APD_50_ ratios ( < 1.4, indicative of a pronounced plateau phase) were classified as ventricular-like, whereas those with high APD_90_/APD_50_ ratios ( > 1.7, indicative of fast phase-I repolarization) were classified as atrial-like. Cardiomyocytes with AP characteristics intermediate between those of the atrial-like and ventricular-like cells, with APD_90_/APD_50_ ratios between 1.4 and 1.7 were classified as nodal-like^14,49–53^. In addition to the above criteria, to categorize hiPSC-CMs into each subtype, action potential morphology was also considered. For example, action potential morphology/phenotype with a negative diastolic membrane potential, a rapid AP upstroke, and a long plateau phase was characteristic of ventricular-like APs. The absence of a prominent plateau phase was a characteristic of atrial-like APs, resulting in shorter AP duration compared to ventricular-like AP. Nodal-like APs showed a more positive MDP, a slower AP upstroke, and a prominent phase 4 depolarization^54^.

## Electronic supplementary material

Supplementary Information

Description of Additional Supplementary files

Supplementary Data 1

Supplementary Data 2

Reporting Summary

## Data Availability

All relevant data is available from the authors. Sequencing data are deposited under the Gene Expression Omnibus (GEO) accession number GSE81585. 10x Genomics sequencing is deposited under synapse ID: syn7818379.
