# Supplementary material for: Defining human cardiac transcription factor hierarchies using integrated single-cell heterogeneity analysis
Source: Nat Commun. 2018 Nov 21;9:4906. doi: 10.1038/s41467-018-07333-4 (PMC6249224; doi:10.1038/s41467-018-07333-4)
Supplement: Supplementary file 1 — Supplementary Information [file 41467_2018_7333_MOESM1_ESM.pdf]

Supplementary Figure 1

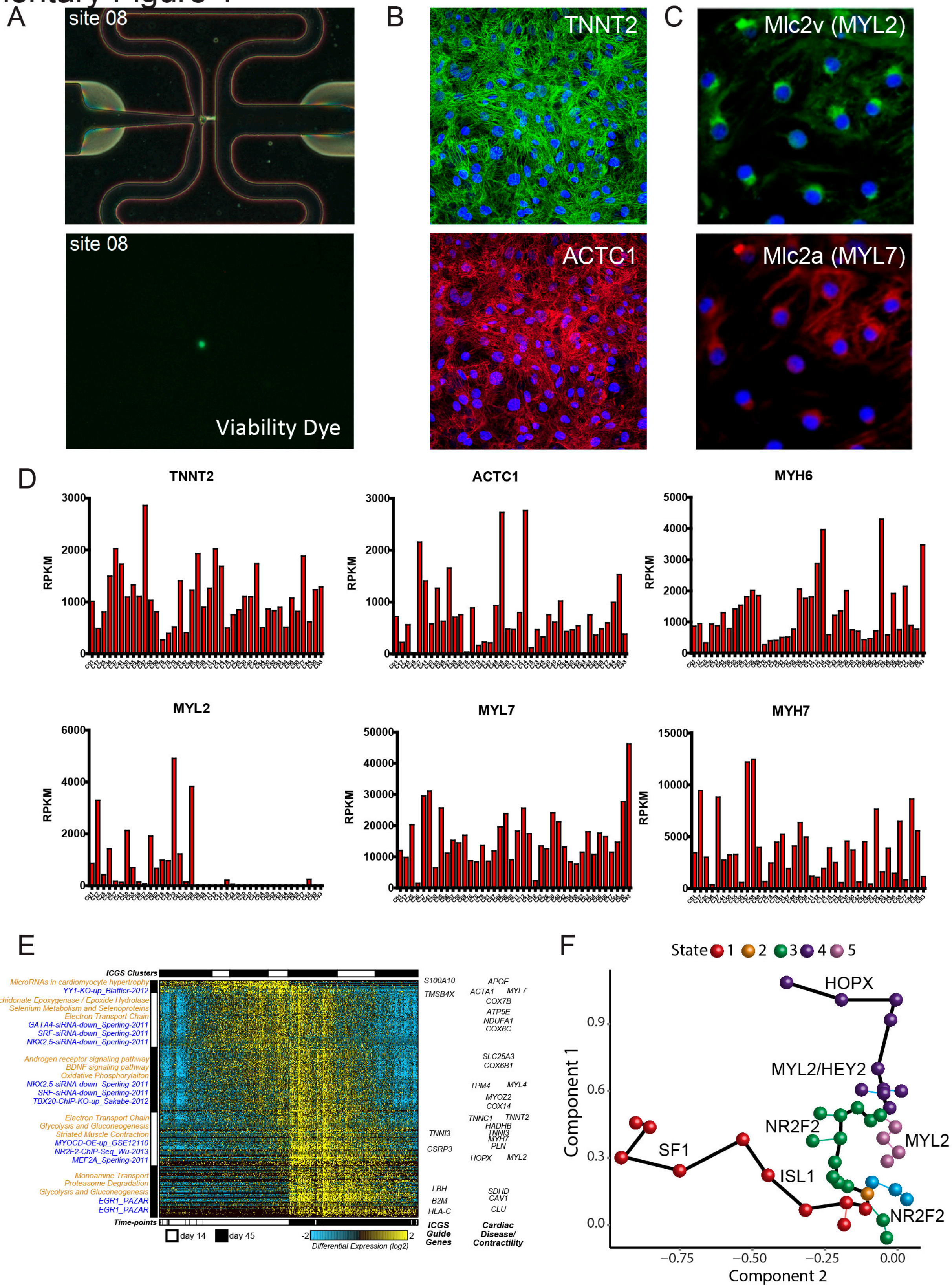

Supplementary Figure 1. Single-cell RNA-seq of hiPSC-CMs using the Fluidigm C1 system. (A) Two hundred hiPSC-CMs at day 30 were run through Fluidigm C1 microfluidic chip to capture single hiPSC-CMs (site 8 shown) and processed for single-cell RNA-seq. Cells were labeled using a viability dye (Calcein-AM) to ensure RNA from live cells were processed. (B, C) Immunofluorescence demonstrated cells expressed known cardiac specific markers (Troponin T Type 2 (TNNT2), Actin, Alpha, Cardiac Muscle 1 (ACTC1), Myosin, Light Chain 7, Regulatory (MYL7) and Myosin, Light Chain 2, Regulatory, Cardiac, Slow (MYL2)). (D) Fifty-four hiPSC-CMs were successfully sequenced which expressed cardiac markers (TNNT2, ACTC1, MYL2, MYL7, MYH6, and MYH7). (E) Single cell 10X genomics RNA-seq clusters called transcription factor and GO terms related to cardiac developmental progression. (F) Monocle applied to single-cell RNA-seq was used to identify a pseudotime progression of different populations of hiPSC-CMs in relation to each other.

Supplementary Figure 2

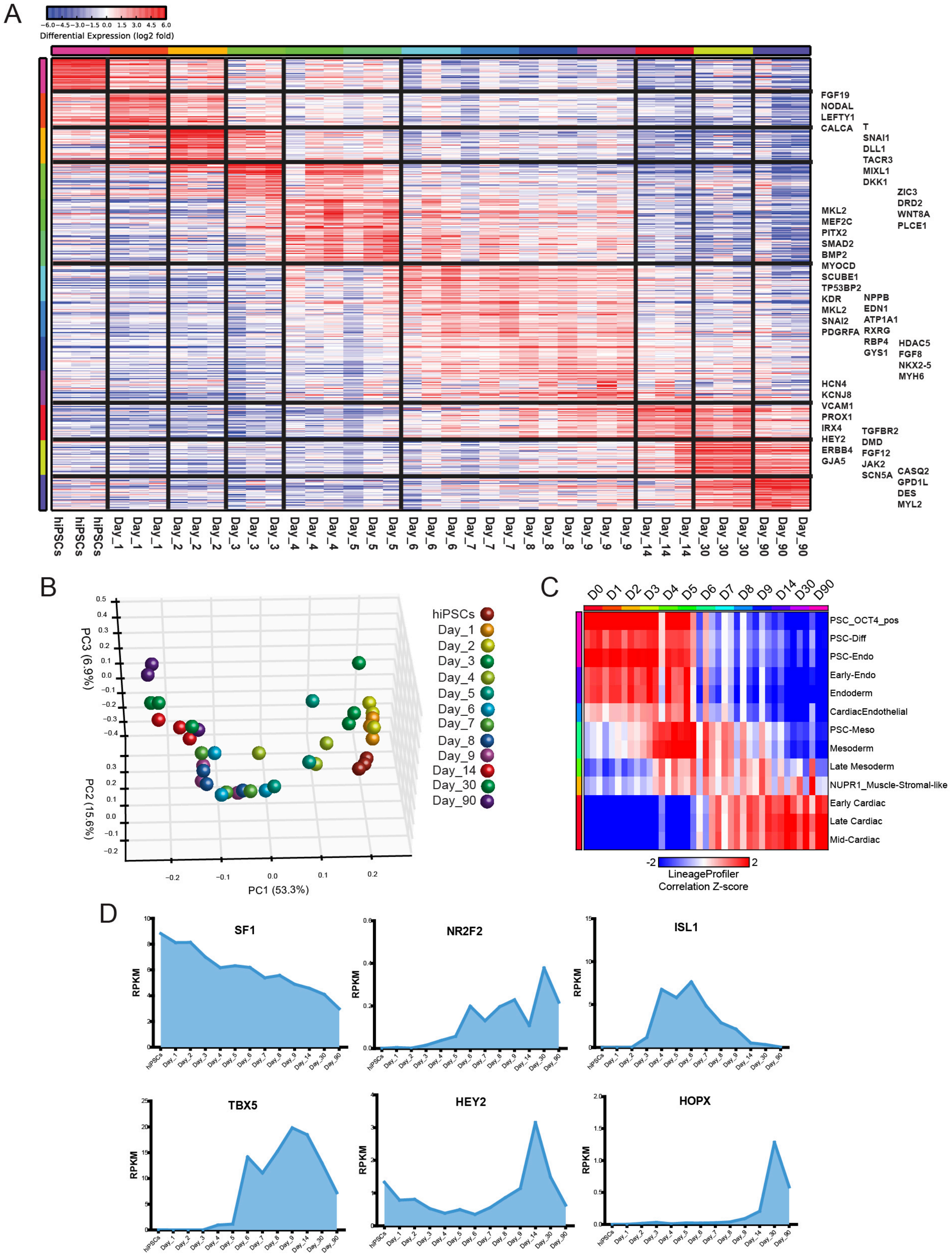

Supplementary Figure 2. Whole transcriptome analysis of differentiation of hiPSCs to hiPSC-CMs. (A) Heatmap of gene markers specific for each day of differentiation. Selected cardiac specific genes are overlaid in the right panel. (B) Principal components analysis (PCA) of RNA-seq expression of hiPSCs undergoing differentiation demonstrates a continuum from hiPSCs to hiPSC-CMs cultured for ninety days. (C) Evaluation of single-cell population heterogeneity among replicate bulk time-course samples is performed using K-nearest neighbor-based classification of bulk RNA-seq time-course samples with the software LineageProfiler. Single-cell population-specific genes (ICGS/MarkerFinder) and expression profiles (Figure 1B) were used to populate the LineageProfiler signature database. Each column represents a distinct bulk RNA-seq sample. Assigned labels for each single cell populations are shown to the right of the heatmap (e.g., PSC_OCT4_pos is a sub-population from the ICGS HOPACH cluster 1). (D) Expression pattern of cardiomyocyte subpopulation markers (SF1, NR2F2, ISL1, TBX5, HEY2, and HOPX) during hiPSC-CM differentiation from bulk time-course RNA-seq data.

Supplementary Figure 3

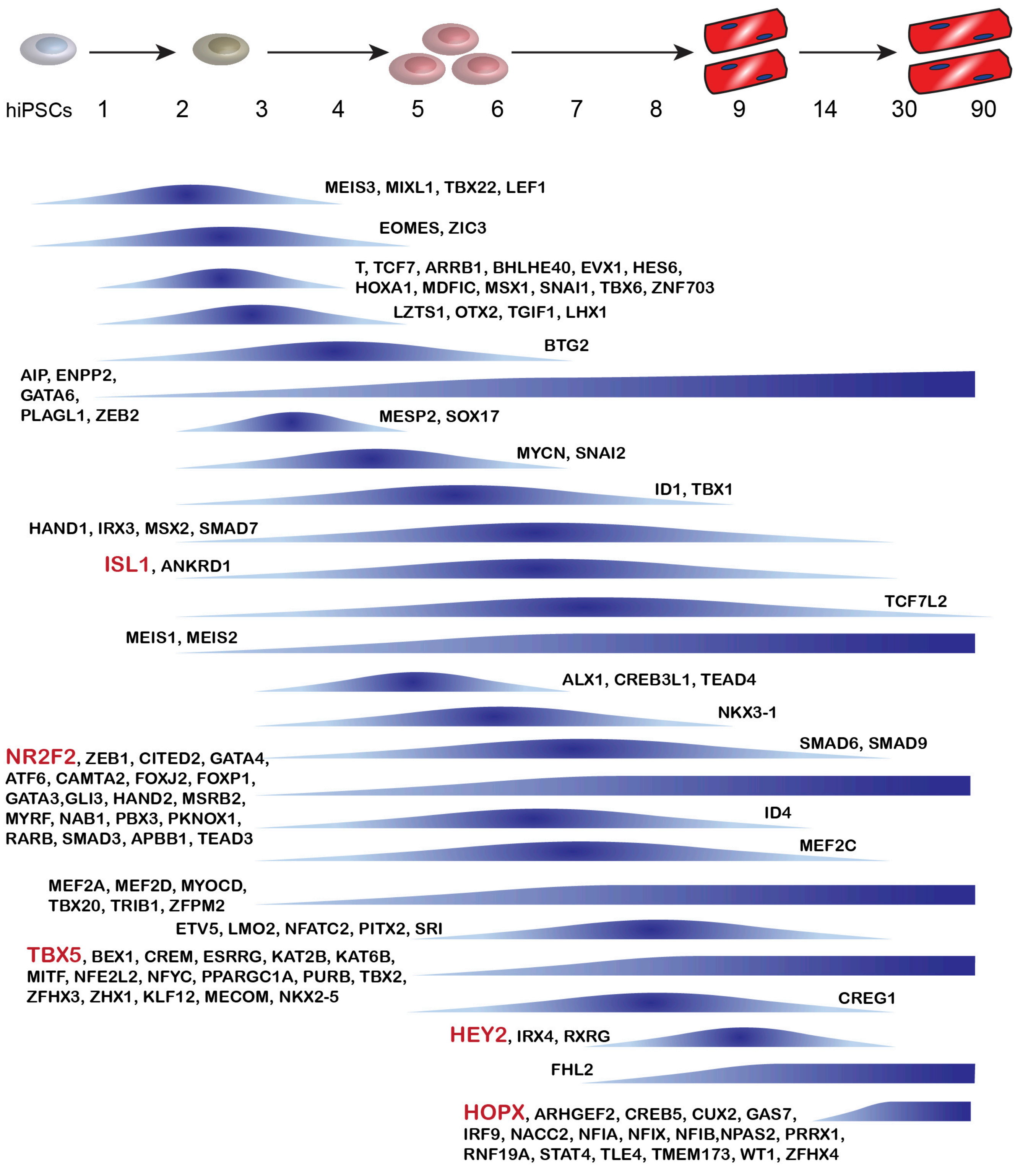

Supplementary Figure 3. Transcription factor hierarchy involved in hiPSC-CM differentiation. RNA-seq expression from each day of differentiation was analyzed for transcription factors demonstrating a greater than two-fold change in expression from each day. A two-fold increase in expression was denoted as the start of transcription factor expression, whereas the corresponding two-fold decrease in expression was denoted as a cessation of expression.

Supplementary Figure 4

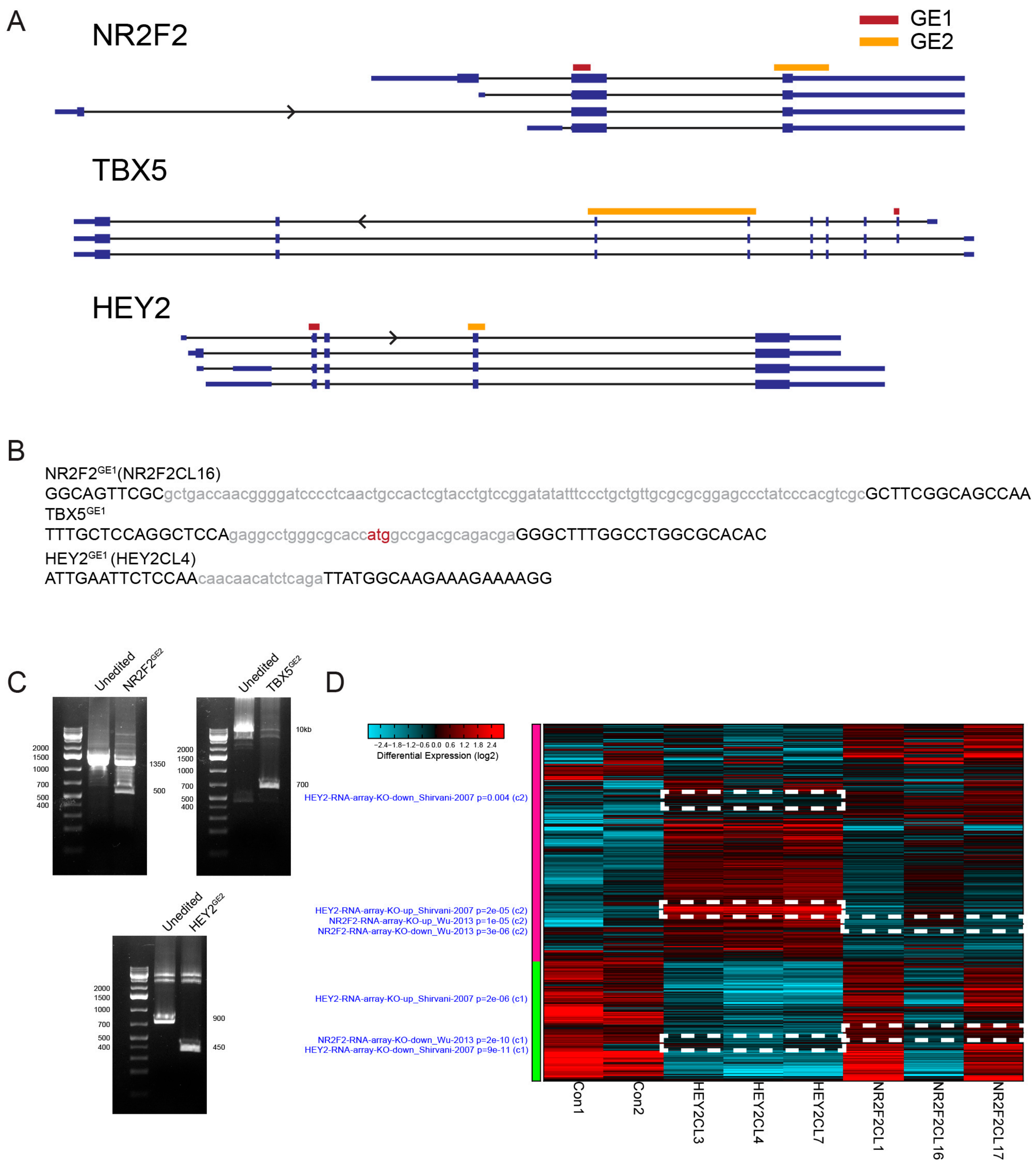

Supplementary Figure 4. Gene editing NR2F2, TBX5, and HEY2 using CRISPR and TALEN. (A) CRISPR guide RNAs were designed against common exonic regions (red line above blue exons) found within multiple transcript variants in NR2F2 and HEY2. In addition, CRISPRs were designed to flank exons to create large deletions spanning whole exons (orange line). (B) Sequencing hiPSC clones identified in genomic regions containing deletions (grey regions) within each gene and annotated as NR2F2GE1, TBX5GE1, and HEY2GE1 gene-edited lines. (C) PCR was performed to demonstrate a heterozygous deletion within NR2F2 (NR2F2GE2) and homozygous exon deletions in TBX5 (TBX-5GE2) and HEY2 (HEY2GE2) lines. (D) Ampli-seq RNA-seq was performed on three putative NR2F2 clones (NR2F2CL1, NR2F2CL16, and NR2F2CL17), three HEY2 clones (HEY2CL3, HEY2CL4, and HEY2CL7), as well as two unedited parental lines (Con1 and Con2). Clustering genes previously published to be either upregulated (left panel containing up) or downregulated (left panel with down) in HEY2 or NR2F2 knockout (KO) conditions with genes differentially expressed from each clone revealed a similar gene expression profile in each HEY2 clone. However, NR2F2CL16 (NR2F2GE1) was the only clone which demonstrated a decrease in genes also published to be down in a NR2F2 KO condition and which expressed higher levels of genes in the NR2F2 condition.

Supplementary Figure 5

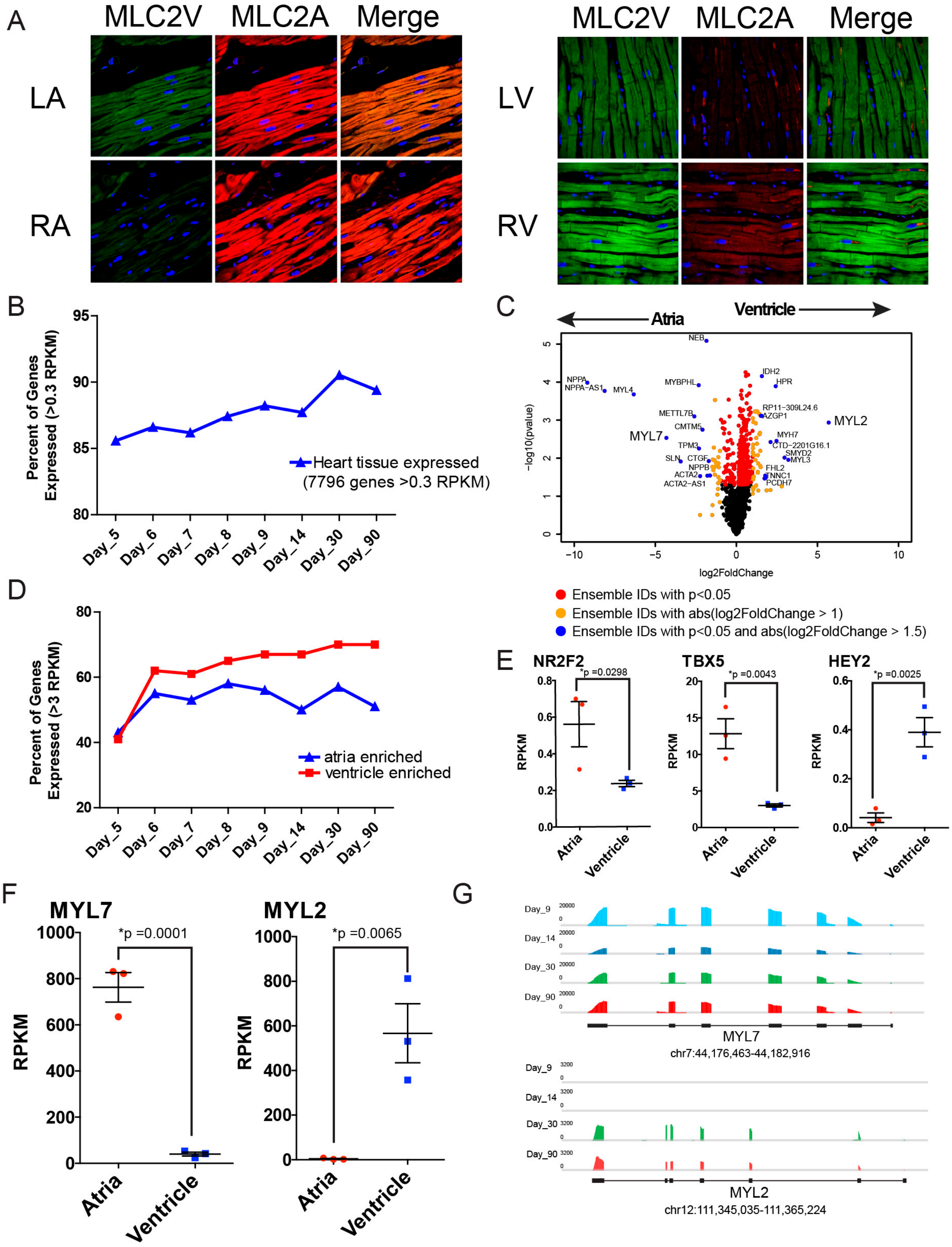

Supplementary Figure 5. Differential expression of atrial and ventricular markers during hiPSC-CM differentiation. (A) Immunofluorescence was performed on each human heart chamber (LA, RA, LV, and RV) using anti-MLC2V and anti-MLC2A antibodies to demonstrate the enrichment of MLC2V within human ventricles and MLC2A within human atria. (B) RNA-seq performed on human heart tissue identified a set of genes that are expressed at greater than 0.3 RPKM (7,796 genes). The percentages of these genes were quantified in hiPSC-CMs during differentiation to evaluate the expression of observed adult heart genes throughout differentiation. (C) Volcano plot was used to visualize atrial or ventricular markers. (D) The percentages of atrial and ventricular markers were assessed during hiPSC differentiation to visualize variation in ventricular enriched marker expression over time. (E, F) Human atria and ventricular expression (RNA-seq) of NR2F2, TBX5, HEY2, MYL2, and MYL7. (G) RNA-seq coverage maps of an atrial marker (MYL7) and ventricular marker (MYL2) during different stages of differentiation.

Supplementary Figure 6

A

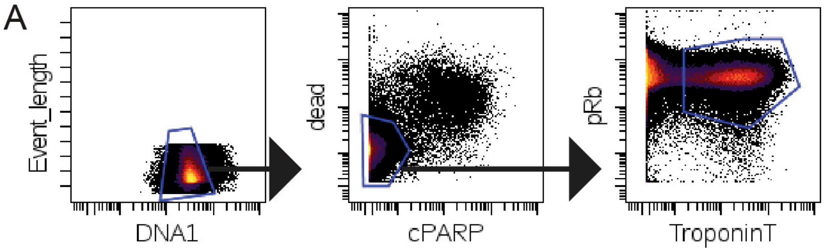

B

Protein	clone	Metal Isotope	Manufacturer
Troponin T	13-11	In113	Thermo Scientific
CD29	TS2/16	In115	Biolegend
pS6(S235/S236)	N7-548	La139	BD Biosciences
CD61	VI-PL2	Ce140	Biolegend
CD326	9C4	Pr141	Fluidigm
CD49e	NKI-SAM-1	Nd142	Biolegend
Tra1-81	TRA-1-81	Nd144	BD Biosciences
CD47	B6H12	Nd145	BD Biosciences
CD117	104D2	Nd146	Biolegend
Beta-catenin	D10A8	Sm147	Fluidigm
pFAK(Y397)	Polyclonal	Nd148	Cell Signaling Technology
CD51	NKI-M9	Sm149	Biolegend
Oct4	40/Oct-3	Nd150	BD Biosciences
MLC2a	56F5	Eu151	Synaptic Systems
pAkt(pS473)	D9E	Sm152	Cell Signaling Technology
GATA4	L97-56	Sm154	BD Biosciences
GFP	FM264G	Gd155	Biolegend
Tra1-60	TRA-1-60	Gd157	BD Biosciences
pStat3(Y705)	4	Gd158	Fluidigm
CD90	5E10	Tb159	Fluidigm
PDGFRalpha	D13C6	Gd160	Fluidigm
Islet1	Q11-465	Dy161	BD Biosciences
CD49d	9F10	Dy162	Biolegend
CD140b	18A2	Dy163	Biolegend
cyclinB	GNS-1	Dy164	Fluidigm
Mlc2v	Polyclonal	Ho165	Protein Tech
Rb(S807/S811)	J112-906	Er166	BD Biosciences
Erk1/2(pT202/pY204)	D13.14.4E	Er167	Fluidigm
Ki67	Ki67	Er168	Fluidigm
CX43	578618	Tm169	R&D Systems
CD49a	TS2/7	Er170	Biolegend
CD49f	GoH3	Yb171	Biolegend
CD31	WM59	Yb172	Biolegend
Total cleaved PARP	F21-852	Yb173	BD Biosciences
VCAM1	51-10C9	Yb174	BD Biosciences
CXCR4	12G5	Lu175	Fluidigm
pHH3	HTA28	Yb176	Biolegend

C

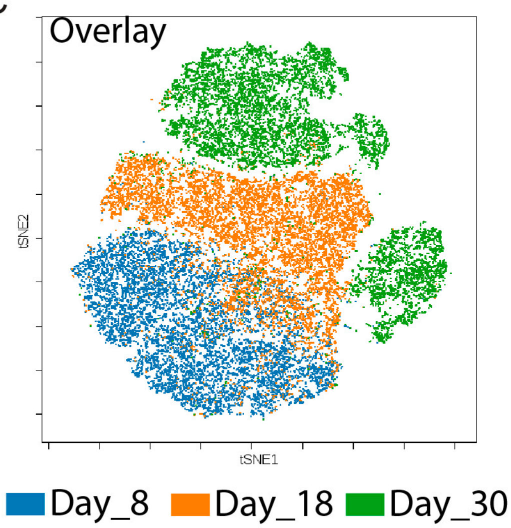

D

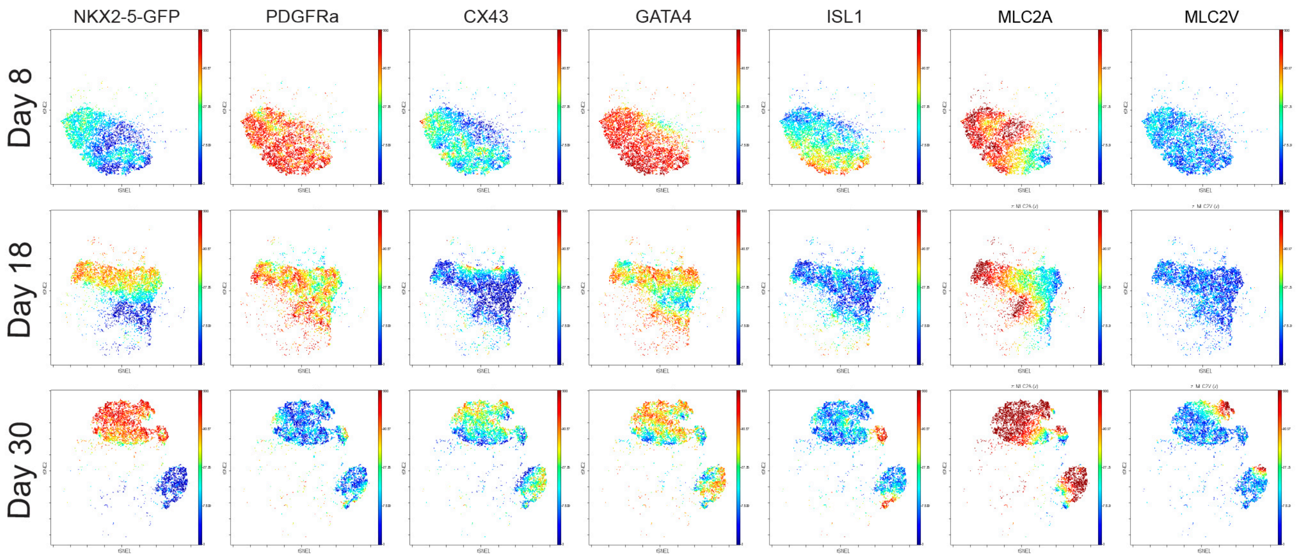

Supplementary Figure 6. Mass cytometry analysis of various cardiac markers of day 30 hESC-CMs. (A) Representative manual gating strategy to define cardiomyocytes. Doublets and debris were first gated out by cell length (Event_length), DNA content (DNA1). Dead and apoptotic cells were excluded based on cisplatin (dead), cleaved product of Poly-ADP-ribose polymerase (cPARP) and phospho-Rb S807/S811 (pRb) staining. Finally, Troponin T was used to define the hESC-CMs. (B) A panel of antibodies used for CyTOF. (C) Overlay of tSNE maps of cardiomyocytes derived from hESCs undergoing cardiac differentiation from day 8 to day 18 to day 30. Each point represents a single cell, and different colors represent samples from different time points. (D) An extended panel of relevant cardiac marker expression patterns (MLC2A, GATA4, GFP, MLC2V, ISL1, PDGFRa, and CX43). Cells are colored based on the intensity of expression of the indicated markers. Higher expression of MLC2A was noted throughout each time point of differentiation, whereas high levels of MLC2V could only be observed at day 30.
