# Supplementary material for: Defining human cardiac transcription factor hierarchies using integrated single-cell heterogeneity analysis
Source: Nat Commun. 2018 Nov 21;9:4906. doi: 10.1038/s41467-018-07333-4 (PMC6249224; doi:10.1038/s41467-018-07333-4)
Supplement: Supplementary file 2 — Description of Additional Supplementary files [file 41467_2018_7333_MOESM2_ESM.pdf]

Supplemental Tables

Supplementary Table 1. Gene expression signatures compiled from transcription factor perturbation transcriptome experiments. Genes were recorded from studies (Experiment) that were reported to change (Genes Defined) in cardiac transcription factor (transcription factor ID: TFID) perturbation or ChIP-seq studies (Method).

Supplementary Table 2. RNA-seq transcriptome of hiPSC-CMs undergoing cardiac differentiation from hiPSCs. RPKM values are reported for each gene during hiPSC-CM differentiation. Each gene is identified by official Hugo nomenclature (Symbol) and RPKM values calculated from each time point assessed (hiPSCs, Day_1, Day_2, Day_3, Day_4, Day_5, Day_6, Day_7, Day_8, Day_9, Day_14, Day_30, and Day_90).
